# Monoterpenes and Sesquiterpenes of Essential Oils from *Psidium* Species and Their Biological Properties

**DOI:** 10.3390/molecules26040965

**Published:** 2021-02-12

**Authors:** Renan Campos e Silva, Jamile S. da Costa, Raphael O. de Figueiredo, William N. Setzer, Joyce Kelly R. da Silva, José Guilherme S. Maia, Pablo Luis B. Figueiredo

**Affiliations:** 1 Programa de Pós-Graduação em Química, Universidade Federal do Pará, Belém 66075-900, Brazil; renan.campos.silva@icen.ufpa.br (R.C.e.S.); joycekellys@ufpa.br (J.K.R.d.S.); gmaia@ufpa.br (J.G.S.M.); 2 Programa de Pós-Graduação em Ciências Farmacêuticas, Universidade Federal do Pará, Belém 66075-900, Brazil; jamile.s.costa@hotmail.com; 3 Centro de Ciência Sociais e Educação, Laboratório de Química, Curso de Licenciatura Plena em Química, Universidade do Estado do Pará, Belém 66050-540, Brazil; raphael.figueiredo@aluno.uepa.br; 4 Department of Chemistry, University of Alabama in Huntsville, Huntsville, AL 35899, USA; wsetzer@chemistry.uah.edu; 5 Aromatic Plant Research Center, 230 N 1200 E, Suite 100, Lehi, UT 84043, USA; 6 Programa de Pós-Graduação em Biotecnologia, Universidade Federal do Pará, Belém 66075-900, Brazil; 7 Programa de Pós-Graduação em Química, Universidade Federal do Maranhão, São Luís 64080-040, Brazil; 8 Departamento de Ciências Naturais, Universidade do Estado do Pará, Belém 66050-540, Brazil

**Keywords:** *Psidium*, Myrtaceae, essential oil variability, monoterpenes, sesquiterpenes, biological activities

## Abstract

*Psidium* (Myrtaceae) comprises approximately 266 species, distributed in tropical and subtropical regions of the world. *Psidium* taxa have great ecological, economic, and medicinal relevance due to their essential oils’ chemical diversity and biological potential. This review reports 18 *Psidium* species growing around the world and the chemical and biological properties of their essential oils. Chemically, 110 oil records are reported with significant variability of volatile constituents, according to their seasonality and collection sites. Monoterpenes and sesquiterpenes with acyclic (C_10_ and C_15_), *p*-menthane, pinane, bisabolane, germacrane, caryophyllane, cadinane, and aromadendrane skeleton-types, were the primary constituents. The essential oils showed various biological activities, including antioxidant, antifungal, antibacterial, phytotoxic, larvicidal, anti-inflammatory, and cytotoxic properties. This review contributes to the *Psidium* species rational and economic exploration as natural sources to produce new drugs.

## 1. Introduction

*Psidium* genus belongs to Myrtaceae, Myrtales order, Rosidae clade, Malvidae subclade, and comprises about 266 species with occurrence in the tropical and subtropical regions of the world. In the Americas, it extends from southern Mexico to Argentina. The centers of high diversity of *Psidium* species are western India, southern Brazil, Paraguay, and northern South America [1,2,3].

*Psidium* species are trees and shrubs, glabrous, glabrescent, or sparsely to densely pubescent; branches cylindrical or tetragonal. Leaves are simple and opposite, exhibiting typical brochidodromous venation; elliptic, cordate, oblong or obovate, chartaceous or coriaceous, glabrous, glabrescent or sparsely to densely pubescent, margin entire, revolute or slightly revolute, midvein slightly sulcate above and prominent below. Flowers are solitary, axillary racemes, dichasium, panicle or inflorescence growing from auxotelic axis producing vegetative shoots with adult leaves after anthesis; floral buds pyriform, entire or constricted above the ovary, round or apiculate, calyx with lobes entirely or partially fused, tearing irregularly at anthesis, 4–5 lobed, pentamerous flowers; calyx cupuliform or calyptrate, petals free, numerous stamens; ovary inferior, with two to five locules and few to many ovules per locule on a peltate to the lamellate intrusive placenta. Fruits with many-seeded, seeds with a bony testa and a cochlear embryo with small cotyledons and a large hypocotyl [4].

*Psidium* species have great ecological relevance. Their fruits are usually fleshly and sought after by several animals, such as birds and mammals, and used as a food source. Thus, this ecological relationship has promoted the conservation of genus diversity since the animals act as seed dispersing agents [5]. Moreover, *Psidium* species can adapt to constant abiotic stress [6], contributing to broad diversification and species’ geographical distribution [7].

Furthermore, *Psidium* species stand out due to the commercial exploitation of edible fruits, essential oils, wood, and plants for ornamental purposes, in addition to presenting pharmacological potential [8]. Traditional communities have empirically used *Psidium* species to treat symptoms of many diseases [9,10]. For example, the tea and infusion of leaves and flowers of *Psidium guajava* L. are used in traditional medicine to treat diarrhea, while the decoction of their roots is used to treat cough, stomach pain, dysentery, toothache, indigestion, and constipation [11,12,13]. Also, the barks’ decoction and poultice are used to treat ulcer wounds, dysentery, skin diseases, vaginal bleeding wounds, fever, dehydration, and respiratory disorders [14]. The decoction of the leaves of *Psidium cattleyanum* Sabine is used to treat stomach pain, and, when associated with *Malva sylvestris* L., *Rubus ulmifolius* Schott, *Elaeagnus umbellata* Thunb., *Allium sativum* L., and *A. cepa* L., it is used against diarrhea [9].

In recent years, reviews have been published referring to essential oils from Myrtaceae’s largest genera, such as *Myrcia, Eugenia*, and *Syzygium* [15,16]. A review of essential oils from Myrtaceae describing various chemical analyses of *Psidium* species has also been published [17]. However, there is no review focusing jointly on the chemical compositions and biological activities of essential oils of *Psidium* species. Due to the chemical and biological significance of *Psidium* species, in this review, there are 110 chemical analyses reported of 18 *Psidium* species growing widespread in the world and their biological activities.

## 2. Bibliographic Search Criteria and Statistical Analyses

Bibliographic research was performed using Google Scholar, PubMed, Science Direct, Medline, and Scopus. Applied keywords were “*Psidium*”, “essential oils”, and “volatile compounds”. Some unusual or incorrect botanical names were updated based on The Plant List” (http://www.theplantlist.org, accessed on 11 February 2021).

Bibliometric data analysis was done using more keywords to search for articles on the theme proposed in this review, using the VOSViewer software (version 1.6.15) [18]. The articles were downloaded from the databases in a supported format by the software. The primary data retrieved from the databases include information related to the article title, authors’ names, keywords, and citation information, including the reference lists. In this way, a cluster was generated relating the main keywords and their links with others used less frequently in the searches.

## 3. Plants Occurrence and the Bibliometric Network Data

**Figure 1:**
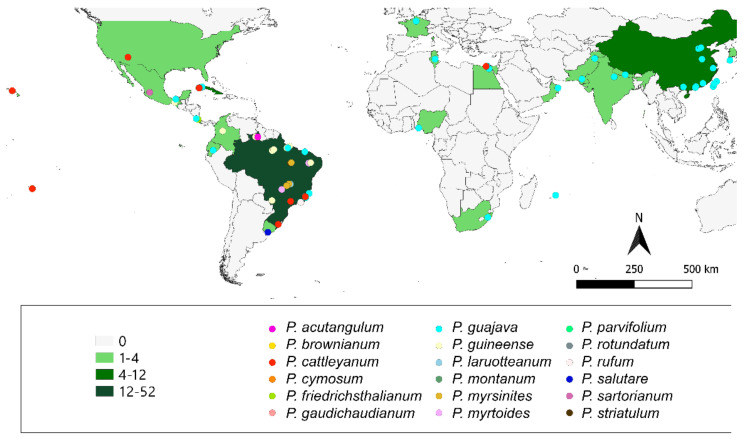
Geographical distribution of *Psidium* based on their essential oils study. This map was built by the authors using the plant occurrence information available in bibliographic data.

Concerning the *Psidium* essential oil reports, 75 are in the Americas: 56 in South America, 14 in Central America, and 5 in North America. Brazil is the most reported country, with 52. Also, 20 reports came from Asia, being half in China. From Africa, 11 reports were obtained, being 4 in Egypt. Only two oils were reported in Europe (France) and Oceania (French Polynesia). The geographical distribution of *Psidium* specimens is shown in Figure 1 and Appendix A.

**Figure 2:**
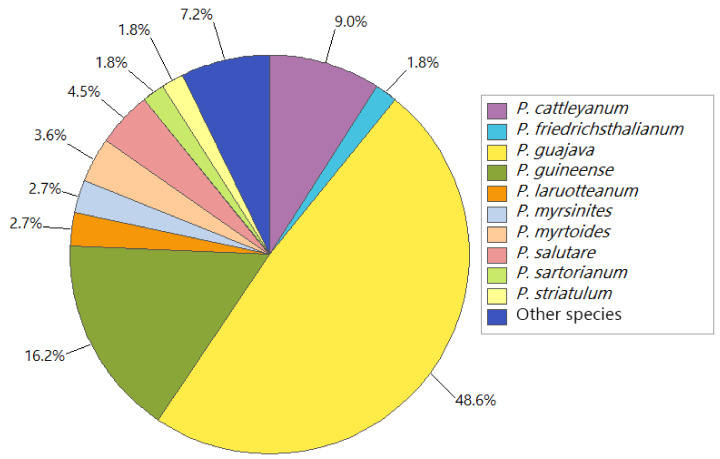
Percentual distribution of the records of essential oils from *Psidium* species.

Regarding the number of reports, *Psidium guajava* L. was the most studied species, with 53 essential oil records (48.6%) distributed in the Americas, Africa, Europe, and Asia. *Psidium guineense* Sw. showed 18 oil records (16.2%), with restricted occurrence in the Americas, and *P. cattleyanum* Sabine with 10 oils records (9.0%) signalized to the Americas and Oceania. Also, five oil records (4.5%) of *P. salutare* (Kunth) O. Berg, four oil records (3.6%) of *P. myrtoides* O. Berg, three oil records (2.7%) of *P. laruotteanum* Cambess and *P. myrsinites* Mart. ex DC., and two oil records (1.8%) of *P. friedrichsthalianum* (O. Berg) Nied., *P. sartorianum* (O. Berg) Nied and *P. striatulum* Mart ex. DC. has been reported only in the Americas. Moreover, the other eight species had only one record (7.2%), *P. acutangulum* Mart. ex DC., *P. brownianum* Mart. ex DC., *P. cymosum* Urb., *P. gaudichaudianum* Proença & Faria, *P. montanum* Sw., *P. parvifolium* (Griseb.) Griseb., *P. rotundatum* Griseb., and *P. rufum* Mart. ex DC. (see Figure 2).

**Figure 3:**
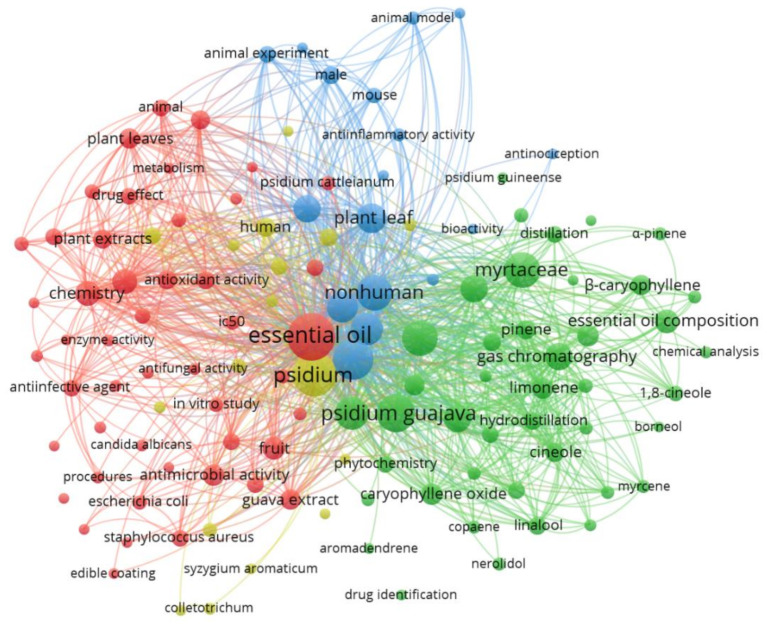
Network map of the most searched keywords and related to the theme, from 1990 to 2020.

To find the most widespread subjects of *Psidium* essential oils and identify their relationships, we analyzed the co-occurrence of similar terms in titles and abstracts of 158 articles in the Scopus database from 1990 to 2020. Figure 3 represents this survey and its associations. The size of the node indicates the extent of searches for the term. That is, the larger the node, the more frequently the term was searched. The search terms are grouped according to their similarity. Thus, the blue cluster includes terms related to in vivo tests, such as “rat”, “animal experiment”, “in humans”, and others. The red cluster contains terms related to biological activities, such as “enzyme activity”, “in vitro study”, “antimicrobial activity”, and “median inhibitory concentration (IC_50_)”. The green cluster shows terms related to chemical composition, such as “essential oil composition”, “chemical analysis”, “hydrodistillation”, “gas chromatography”, “cineole”, “limonene”, etc. Finally, in yellow and to a lesser extent, one can observe a cluster four with terms related to this article’s theme.

## 4. Volatile Profiles

The essential oils’ chemical profile is influenced by environmental factors, contributing to their primary and secondary constituents’ chemical variability. This fact takes along several chemical profiles, chemotypes, or different chemical races to the same species. Therefore, it is worth considering that minor constituents in essential oils can play an essential role in the chemical polymorphism of a given species [19,20].

**Figure 4:**
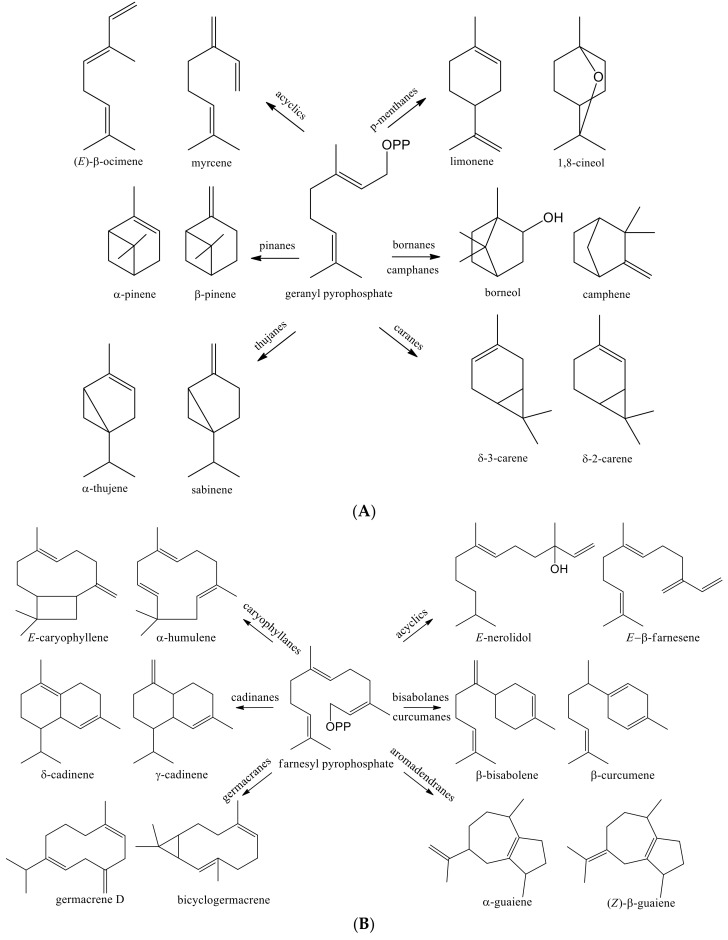
Representative chemical skeletons of geranyl (**A**) and farnesyl (**B**) pyrophosphate pathways of some sesquiterpenes found in the essential oils of *Psidium* species.

The essential oils of *Psidium* species are rich in mono- and sesquiterpenes compounds arranged according their biosynthetic pathways [21,22,23,24,25]. The C_10_-skeletal monoterpenes are grouped in acyclic, *p*-menthane, pinane, bornane/camphane, thujane, and carane types, and the C_15_-skeletal sesquiterpenes are arranged as acyclic, cadinane, caryophyllane, eudesmane, germacrane, aromadendrene, and bisabolane types [26,27] (see Figure 4A,B and Appendix B).

### 4.1. Psidium cattleyanum Sabine

It is native to Brazil, occurring from Minas Gerais to the Rio Grande do Sul states and Uruguay northeastern, and as an isolated form in the Amazon region. It was also naturalized in tropical climates regions such as Hawaii and many Caribbean islands. Commonly, it is known as strawberry guava, bovine guava, jeju guava, cherry guava, purple guava, waiawi, guava, and araçá [28,29]. In some reports, its name has been published as *Psidium cattleianum*, probability by a written error [30,31]. *Psidium cattleyanum* fruits are similar to *P. guajava* (guava); however, with a slightly acid flavor. For this reason, they are widely consumed in nature or juices, jellies, and ice cream forms, with good consumer acceptance and high value for the agri-food industries [32,33]. The vitamin C content in *P. cattleyanum* fruits is around 3 to 4 times higher than lemon and orange [34,35]. Furthermore, this species has been used in traditional medicine for several purposes, such as antiseptic, digestive, anti-hemorrhagic, blood pressure control, and diuretic [32].

The variability of EO composition of ten accessions of *P. cattleyanum* (nine from leaves and one from fruits) were classified into different chemical profiles. In all samples, at least one constituent with caryophyllane skeleton was identified. Three profiles of leaves oils were characterized only by caryophyllane-type constituents: *E*-caryophyllene (31.5%) in profile I from French Polynesia [36]; *E*-caryophyllene (59.9%) and caryophyllene oxide (5.4%) in profile II from USA [37]; *E*-caryophyllene (59.6%), caryophyllene oxide (18.2%) and *Z*-caryophyllene (6.4%) in profile III from south Brazil [38]. Profile IV oil from the USA was rich in *E*-caryophyllene (59.0%), followed by acyclic monoterpenes, such as α-pinene (13.2%) and myrcene (11.3%), respectively [39]. Profile V oil from southeastern Brazil was characterized by the *E*-caryophyllene (23.4%), caryophyllene oxide (11.5%), and α-pinene (11.3%) [22]. Profile VI oil from Egypt was rich in *E*-caryophyllene (28.8%), α-pinene (28.0%), myrcene (13.4%), and trans-β-ocimene (5.3%) [40].

Fruits oil from south Brazil, classified as profile VII, was rich in skeletons of the types caryophyllane (*E*-caryophyllene 22.5% and α-humulene 7.5%), eudesmane (neo-intermedeol 14.2% and β-selinene 10.1%), and aromadendrene (*trans*-β-guaiene, 9.1%) [41]. Sesquiterpenes dominated profile VIII oil from Cuba with cadinane and caryophyllane skeleton types, such as *epi*-α-muurolol (21.9%), α-cadinol (20.0%), *epi*-α-cadinol (16.7%), caryophyllene oxide (13.6%), juniper camphor (9.4%), and 14-hydroxy-9-*epi*-*E*-caryophyllene (5.7%) [42]. Profile IX oil from Brazilian Atlantic Forest was characterized by skeletons of the types thujane (α-thujene 25.2%), *p*-menthane (1,8-cineole 16.4%), caryophyllane (*E*-caryophyllene 10.2%), eremophilane (valencene, 8.0%), and acyclic monoterpene (myrcene, 5.0%) [43]. Also, the profile X oil from Brazilian Midwest was rich in the types aromadendrane/eudesmane (viridiflorol 17.9%, β-selinene 8.6% and aromadendrene 5.0%), caryophyllane (*E*-caryophyllene 11.8% α-humulene 6.0%), and *p*-menthane (1,8-cineole 10.8%) [44].

### 4.2. Psidium friedrichsthalianum (O. Berg) Nied

It is a fructiferous plant native from forests and savannas of higher regions from Honduras to Panama, also found in Colombia mountains and widely cultivated in Costa Rica by the trivial names ‘cas’ and ‘goiaba costarriquenha’ [45,46]. The fruits can be consumed as refreshing drinks, jellies, and jams due to its acidity and filling for pies [46,47].

Two oils from *P. friedrichsthalianum* leaves showed different chemical profiles: a Costa Rica specimen with caryophyllane, elemane, pinane, germacrane, and cadinane skeletons, represented by *E*-caryophyllene (36.8%), β-elemene (12.86%), α-pinene (10.6%), bicyclogermacrene (8.3%), β-pinene (8.3%), and α-ylangene (7.8%) [37]; and a Southeast Brazil specimen with the caryophyllane (*E*-caryophyllene 24.6%, caryophyllene oxide 10.6%, and α-humulene 9.2%) and cadinane (α-5.9%) types [22].

### 4.3. Psidium guajava L.

*Psidium guajava*, commonly known as ’guava‘, is native to tropical regions from southern Mexico to northern South America. It is a species cultivated in many countries, and this fact allows its reproduction on a large scale in tropical and subtropical climates [48]. In folk medicine, this species is used as tea, infusion, decoctions, and poultice to treat inflammation, diarrhea, rheumatism, and diabetes, and as a diuretic, anti-bacterial [48]. Moreover, its fruits can be consumed in nature or sweets, ice cream, and juices forms, so this species has the highest commercial value of all *Psidium* species [28].

Fifty-three records of *Psidium guajava* essential oils were arranged in 28 profiles based on the chemical skeleton of their main compounds. The profile I was characterized by sesquiterpenes with caryophyllane, acyclic and aromadendrene types. Primary compounds were *E*-caryophyllene (15.8–24.6%), *E*-nerolidol (7.7–35.6%), caryophyllene oxide (5.1%), (2*Z*,6*E*)-farnesol (6.7%), and ledol (5.5%) of oil records from Asia (Pakistan) and Europe (France) [49,50,51]. Profile II corresponded to a record oil from Cuba, rich in the sesquiterpenes of caryophyllane, acyclic, cadinane and aromadendrene types: *E*-caryophyllene (21.6%), *E*-nerolidol (l9.2%), selin-11-en-4α-ol (13.4%), viridiflorene (8.8%), α-selinene (8.3%), caryophyllene oxide (8.2%), and cedr-8(15)-en-9α-ol (7.9%) [52]. The profile III displayed compounds with caryophyllane and aromadendrene skeletons, as *E*-caryophyllene (24.4–36.4%) and γ-gurjunene (12.7–14.0%) corresponding to three oil records from China [53], and another from Tunisia, which was characterized by viridiflorol (36.4%) and *E*-caryophyllene (5.9%) [54].

Profile IV was composed by sesquiterpenes with caryophyllane, cadinane, and aromadendrene skeletons, as *E*-caryophyllene (17.2–25.7%), calamenene (6.6–7.8%), γ-gurjunene (9.2–9.5%), and *epi*-α-cadinol (6.0–10.0%) occurring in five oil records from Asia (China and Taiwan) [53]. Profile V was composed by three oil records from Brazil, belonging to caryophyllane, bisabolane, *p*-menthane, and acyclic sesquiterpene types, as *E*-caryophyllene (5.2–9.4%), β-bisabolol (9.2–19.5%), limonene (0–17.8%), 1,8-cineole (2.0–21.4%), *E*-nerolidol (5.0–7.4%), α-humulene (0–16.5%), β-bisabolene (0–6.3%), humulene epoxide II (0–6.0%), and α-pinene (0–23.9%) [55,56]. Profile VI from china was characterized by constituents with caryophyllane (*E*-caryophyllene, 26.4%), bisabolane (β-bisabolene, 5.23%), and aromadendrene (γ-gurjunene, 15.2%) skeletons [53]. Profile VII from Egypt was composed by skeleton-types of caryophyllane (*E*-caryophyllene, 16.9%), eudesmane (selin-7(11)-en-4-α-ol, 8.3%, α-selinene, 6.5%, β-selinene, 6.3%), cadinane (δ-cadinene, 5.3%), and *p*-menthane (1,8-cineole, 5.4%) [57].

Profile VIII from Brazil displayed caryophyllane and aromadendrane skeleton types, as *E*-caryophyllene (26.6%), selin-11-en-4α-ol (6.7%), caryophyllene oxide (15.5%), aromadendrene epoxide (8.1%), β-selinene (7.6%), and α-selinene (6.5%) [55]. Profile IX from Brazil was composed by caryophyllane (*E*-caryophyllene 19.4%, caryophyllene oxide 16.6%), selinane (selin-11-en-4α-ol 7.4%, β-selinene 5.6%), *p*-menthane (1,8-cineole 8.4%), and aromadendrene (aromadendrene epoxide 9.2%) skeleton types [55]. Profile X composed by oil records from Brazil and Polynesia, showed sesquiterpenes with caryophyllane (*E*-caryophyllene 10.2–39.0%, caryophyllene oxide 0–8.0%), selinane (selin-11-en-4α-ol 0–16.7%, α-selinene 4.8–9.7%, β-selinene 4.1–9.7%), aromadendrene (aromadendrene epoxide 0–9.5%, aromadendrene 0–6.3%), cadinane (epi-α-cadinol 0–7.9%, *epi*-cubenol 0–6.7%), *p*-menthane (1,8-cineole 0.3–7.6%), and bisabolane (*E*-α-bisabolene 0–5.5%) skeleton types [36,55,58,59].

Profile XI was recorded in the Americas (Cuba), Asia (Taiwan, Korea, India), and Africa (Nigeria, Egypt Mauritius) and characterized by mono- and sesquiterpenes with caryophyllane, aromadendrane, and *p*-menthane skeletons, as *E*-caryophyllene (6.8–21.3%), α-pinene (0–14.7%), 1,8-cineole (0–12.4%), aromadendrene (0–6.6%), limonene (0–42.1%), α-selinene (0–6.6%), β-selinene (0–6.4%), caryophyllene oxide (0–15.4%), caryophylla-4(12),8(13)-dien-5β-ol (0–6.5%) [57,60,61,62,63,64,65]. Profile XII was composed by an oil record from Asia (Nepal), characterized by caryophyllane, acyclic, and cadinane skeleton types, as caryophyllene oxide (14.0%), *E*-caryophyllene (13.9%), 1H-cycloprop[e]azulene (11.7%), adamantane (9.5%), *E*-nerolidol (6.8%), and α-cubebene (6.7%) [66]. Profile XIII from Brazil was characterized by caryophyllane (α-humulene 15.0%, *E*-caryophyllene 12.0%), selinane (β-selinene 11.0%, α-selinene 10.0%), cadinane (α-muurolol 5.6%), and cedrane (cedr-8-(15)-en-9-α-ol 7.6%) skeleton types [67].

Profile XIV from oil records from Brazil and Tunisia was characterized by sesquiterpenes with caryophyllane, germacrane, eudesmane, cadinane, and aromadendrene skeletons, as germacrene D (0–16.8%), α-humulene (13.0–15.0%), caryophylla-4(12),8(13)-dien-5-β-ol (0–15.0%), *E*-caryophyllene (0–12.0%), valerianol (0–10.6%), α-selinene (0–10.0%), α-muurolol (0–9.6%), cedr-8(15)-en-9-α-ol (0–7.6%), β-selinene (0–6.7%), humulene epoxide II (0–6.6%), *epi*-α-muurolol (0–5.6%), caryophyllene oxide (0–5.1%), and α-cadinol (0–5.0%) [54,67,68]. Profile XV from Brazil was dominated by caryophyllane and eudesmane skeletons, as α-humulene (37.0%), *E*-caryophyllene (24.0%), β-selinene (14.0%), and α-selinene (12.0%) [67]. Profile XVI from China was rich in mono- and sesquiterpenes with *p*-menthane, pinane, aromadendrane and cedrane skeletons, as α-pinene (37.8%), 1,8-cineole (18.9%), globulol (6.8%), and cedr-8(15)-en-9-α-ol (5.6%) [69]. 

Profile XVII from China was characterized by monoterpenes with *p*-menthane, pinane and carane skeletons (α-pinene 25.5%, δ-3-carene 8.8%, limonene 9.8%), followed by sesquiterpenes with acyclic, caryophyllane, cedrane and aromadendrane skeletons, as *E*-nerolidol (16.7%), *E*-caryophyllene (15.7–33.5%), cedran-8-ol (8.8%), viridiflorene (13.0%), and (*E*)-β-farnesene (11.7%) [70,71]. Profile XVIII from oil records of Ecuador and Egypt was characterized by monoterpenes with *p*-menthane and pinane skeletons as: limonene (33.3–54.7%), α-pinene (1.5–29.5%), carvotacetone acetate (0–8.2%) and 1,8-cineole (0–32.1%) [40,72]. The profile XIX from Cuba was characterized by the monoterpene limonene (8.3%) and 3-phenylpropylbenzoyl acetate (6.2%) [61]. 

Profiles XX–XXV and XXVII–XXVIII were registered to only one *P. guajava* sample from different locations. Profile XX from Mauritius was characterized by bisabolane (santalol 50.6%), caryophyllane (caryophyllene oxide 15.4%), *p*-menthane (limonene (11.6%), and cadinane (cycloisosativene, 6.1%) skeleton types [73]. Profile XXI from Costa Rica was dominated by 2*E*-hexenal (28.4%) and benzaldehyde (16.5%), followed by the sesquiterpene types *p*-menthane (1,8-cineole 15.9%), aromadendrene (globulol 10.3%), and acyclic (*E*-nerolidol 6.9%) [74]. Profile XXII from France, extracted by HS-SPME, was also ruled by other compounds, as hexanal (65.9%), γ-butyrolactone (7.6%), and 2*E*-hexenal (7.4%) [49]. The fruits volatile concentrate from Cuba, obtained by simultaneous distillation-extraction (SDE), was classified as profile XXIII, rich in a mixture of other classes of constituents, with 3*Z*-hexenyl acetate (5.0%) as the major compound [61]. Profile XXIV from Mexico specimen leaves displayed bisabolane-skeleton constituents, as β-bisabolene (19.2%), β-sesquiphellandrene (14.8%), *E*-γ-bisabolene (5.3%), and α-curcumene (5.1%), followed by *E*-caryophyllene (6.0%) [37]. Profile XXV of fruits concentrate from Cuba showed *E*-cinnamyl acetate (5.6%) as the most significant constituent [61]. Profile XXVII from Pakistan was rich in hexanol (13.9%), cinnamyl alcohol (10.9%), butanol (10.7%), 3-methyl glutaric anhydride (9.5%), hexene (7.7%), butanoic acid methyl ester (7.2%), and 3-hexenal (6.6%) [75]. Profile XXVIII from South Africa specimen leaves was characterized by 4,4-dimethyl-tetracyclo [6.3.2.0(2,5).0(1,8)]tridecan-9-ol (13.0%) and 1H-cycloprop[e]azulene (8.1%), followed by *E*-caryophyllene (9.6%) [66].

Profile XXVI was composed of leaves oils from 22 genotypes of *P. guajava* grown in Espírito Santo state, Brazil, at two different environments and showed a great chemical diversity. The main constituents were sesquiterpenes with acyclic, caryophyllane, bisabolane, cadinane, germacrene, and aromadendrene skeletons, followed by *p*-menthane monoterpene constituents. Oil samples from the city of Mimoso do Sul displayed *E*-caryophyllene (5.1–30.0%), α-humulene (2.0–24.4%), 14-hydroxy-*epi*-*E*-caryophyllene (1.3–19.3%), β-bisabolol (1.2–20.1%), *E*-nerolidol (0.5–19.9%), 14-hydroxy-*epi*-*E*-caryophyllene (0–14.7%), limonene (0.2–11.7%),γ-muurolene (1.5–6.4%), α-selinene (0.4–12.4%), β-selinene (0.5–13.3%), β-bisabolene (3.1–9.7%), hinesol (0.9–10.0%), *epi*-α-cadinol (0–6.4%), α-bisabolol (1.0–5.9%), selina-6-en-4-ol (0.6–9.1%), aromadendrene (0.3–7.4%), and 1,8-cineole (0.7–5.3%) as the primary constituents. Oils samples from the city of Linhares showed *E*-caryophyllene (5.1–32.3%), caryophyllene oxide (1.8–20.9%), α-humulene (1.7–19.9%), β-bisabolol (2.2–19.4%), *E*-nerolidol (2.1–13.7%), hinesol (3.2–12.4%), α-selinene (0.5–11.2%), b-selinene (0.5–12.8%), *epi*-α-cadinol (1.1–12.0%), limonene (0.1–11.0%), β-bisabolene (2.3–9.7%), α-bisabolol (0.5–7.3%), *epi*-β-cubenol (2.2.–7.1%), humulene epoxide (0.6–6.3%), selina-6-en-4-ol (3.3–6.1%), aromadendrene (3.1–5.6%), γ-muurolene (1.7–5.4%), and δ-cadinene (0.5–5.1%) as the major components [24].

### 4.4. Psidium guineense Sw.

It is a native species of South America, from northern Argentina throughout the Brazilian territory and in isolation form in southern Mexico. Commonly known as ‘araçá’, ‘araçá-comum’, ‘araçá-azedo’, and ‘araçá-do-campo’ [28,76]. *Psidium guineense* (syn. *Psidium araca* Raddi or *P. guianense* Pers., due to a written error that gave it an African name despite its American origin) presents a significant economic and medicinal potential exploitation [77,78]. *Psidium guineense* fruits have a high content of minerals and functional elements, such as vitamins and phenolic compounds, consumed freshly or used to prepare sweets, juices, ice cream, and jellies [79,80,81]. In folk medicine, all parts of “araçá” are widely used in South America to treat gastrointestinal and genitourinary infections [82].

Eighteen accessions of leaves (16 records) and fruits (2 records) oils of *P. guineense* were classified in 16 chemical profiles, according to the major constituents (>5%), corresponding to monoterpenes with *p*-menthane and pinane skeletons, followed by sesquiterpenes with acyclic, bisabolane, germacrane, elemane, caryophyllane, and cadinane skeletons. Profiles I–V, VII–IX, and XI–XIII occur in Northern Brazil, profile VI in Northeast Brazil, profile X in Mexico, profile XIV in South Brazil, all of them from leaves, and profiles XV and XVI from fruits occur in Colombia. Profile I was dominated by limonene (47.4%) [20]; Profile II by limonene (30.4–37.2%) and α-pinene (17.7–34.0%) [21]; Profile III by limonene (26.5%), α-pinene (13.7%), and α-copaene (7.2%) [21]; Profile IV by limonene (9.6%) and *epi*-β-bisabolol (6.5%) [21]; Profile V by limonene (23.4%), *epi*-β-bisabolol (9.5%), and β-bisabolene (6.4%) [21]; Profile VI by 1,8-cineole (40.5%), β-eudesmol (19.5%), α-pinene (13.9%), β-pinene (8.6%), elemol (7.7%), and γ-eudesmol (5.2%) [83]; Profile VII by α-pinene (35.6%), α-copaene (8.1%), *E*-caryophyllene (6.1%), and muurola-4,10(14)-dien-1-β-ol (5.8%) [21]; Profile VIII by α-pinene (26.4%), limonene (14.0%), and *E*-caryophyllene (5.2%) [21]; Profile IX by β-bisabolene (8.9%) and *α*-curcumene (5.0%) [21]; Profile X by β-bisabolene (13.2%), α-pinene (12.5%), *Z*-nerolidol (5.5%), β-sesquiphellandrene (5.2%), and limonene (5.1%) [37]; Profile XI by *E*-caryophyllene (24.0%) and limonene (5.4%), [21]; Profile XII by β-bisabolol (17.4%), limonene (6.8%), and *epi*-α-bisabolol (6.7%) [56]; Profile XIII by *epi*-β-bisabolol (18.1%) and β-bisabolol (5.6%) [21]; Profile XIV by spathulenol (80.7%) [84]. The two oils of *Psidium guineense* fruits showed different chemical compositions. Profile XV extracted by SDE showed *E*-caryophyllene 8.6%, butanol 7.4%, ethyl butyrate 7.4%, and selin-11-en-4α-ol 5.9% as the main constituents. Profile XVI extracted by HS-SPME exhibit ethyl butyrate 30.3% and ethyl hexanoate 23.8% [85].

### 4.5. Psidium laruotteanum Cambess

The species occurs in Central Brazil, and it is commonly known as ‘araçá-cascudo’ Its fruits are similar to the gooseberry, showing a more acidic flavor than ‘araçá’. It is consumed in nature by wild animals and as jams and juice forms by people. This plant has low fruit productivity [30,86,87].

Regarding the chemical variability of leaves essential oils of *Psidium laruotteanum*, the records from Midwest Brazil were classified into three chemical profiles, rich in monoterpenes with *p*-menthane and pinane skeletons. Profile I was rich in *p*-cymene (24.8%), 1,8-cineole (19.2%), α-pinene (13.4%), and terpinen-4-ol (6.3%) [86]. Profile II was dominated by *p*-cymene (19.4%), α-pinene (11.6%), 1,8-cineole (6.9%), terpinen-4-ol (5.8%), and followed also by γ-terpinene (14.0%), limonene (10.2%), and terpinolene (5.1%) [86]. Profile III was characterized by *p*-cymene (34.8%), 1,8-cineole (12.5%), α-pinene (9.2%), limonene (7.9%), γ-terpinene (6.9%), and α-terpineol (6.0%), as primary constituents [86]. The three chemical profiles showed quantitative and qualitative variability, probably due to the specimens being collected in the wild and not subjected to the same conditions of environmental control, except in their vegetative stages, without flowers and fruits.

### 4.6. Psidium myrsinites DC

Native from Brazil, it is also known as ‘araçá’, and the fruits are used in folk medicine for cauterization and to treat diarrhea due to its astringent properties [28,88]. 

Three *P. myrsinites* records showed quantitative and qualitative differences in the chemical composition of their leaves’ essential oils, with the predominance of caryophyllane-type skeletons.

Profile I from Goiás (Midwest Brazil) showed *E*-caryophyllene (31.0%), α-humulene (12.3%), and caryophyllene oxide (7.3%) as the major constituents [88]. Profile II from Maranhão (Northeast Brazil) was rich in *E*-caryophyllene (26.1%), 𝛼-humulene (23.9%), caryophyllene oxide (10.1%), humulene epoxide II (6.4%), and Caryophylla-4(12),8(13)-dien-5-β-ol (5.7%) [89]. Profile III from Federal District (Midwest Brazil), displayed caryophyllene oxide (26.1%), humulene epoxide II (8.8%), *E*-caryophyllene (7.4%), *Z*-caryophyllene (5.4%), and myrcene (5.4%) as the main constituents [90].

### 4.7. Psidium myrtoides O. Berg

*Psidium myrtoides* (syn. *Psidium myrsinoides* Berg) occur in all Brazilian territory, and it is commonly known as ‘araçá-de-veado’ (deer aracá) or just ‘araçá’ [23,30,91]. In folk medicine, as other ‘araçá’ species, *P. myrtoides* is also used to treat diarrhea [92].

*Psidium myrtoides* leaf essential oils recorded in some Brazilian states were classified into three chemical profiles, according to their primary constituents. These oil records were rich in mono- and sesquiterpenes with acyclic, *p*-menthane, pinane, caryophyllane, germacrane, elemane, cadinane, and bisabolane skeleton types. Profile I from Federal District (Midwest Brazil) showed *E*-caryophyllene (22.4%), caryophyllene oxide (19.7%), α-humulene (8.4%), and myrcene (5.4%) as the main constituents [23]. Profile II from Ceará state (Northeast Brazil) was dominated by 1,8-cineole (29.9–48.1%), α-eudesmol (11.7–20.0%), α-pinene (5.0–12.8%), elemol (3.3–6.0%), and γ-eudesmol (2.5–5.8%) [93]. Profile III, composed of two oil records from Goiás state (Midwest Brazil) and Espírito Santo state (Southeastern Brazil), showed the sesquiterpenes *E*-caryophyllene (19.4–30.9%), α-humulene (10.4–15.9%), α-bisabolol (7.3–10.4%), α-copaene (6.3–7.8%), and caryophyllene oxide (5.3–7.3%) as the primary components [22,91].

### 4.8. Psidium salutare (Kunth) O. Berg

It is widely distributed in South America, known as ‘araçá’, ‘araçá-da-pedra’, and ‘araçá-do-campo’ in Brazil, ‘guayabo arrayan’ and ‘managuá’ in the Dominican Republic, and guayabita in Cuba [28,94]. *Psidium salutare* [syn. *P. incanum* (O. Berg) Burret and *P. luridum* (Spreng.) Burret]. Also, four varieties were described to this species: *P. salutare* var. *sericeum* (Cambess.) Landrum, *P. salutare* var. *mucronatum* (Cambess.) Landrum, *P. salutare* var. *decussatum* (DC.) Landrum, and *P. salutare* var. *pohlianum* (O. Berg) Landrum, with occurrence in Paraguay, the Caribbean, and Mexico [95].

Five *Psidium salutare* leaf oil records were classified into 4 chemical profiles. Constituents characterized the first profile from Uruguay with *p*-menthane and acyclic skeletons, as 1,8-cineole (31.2–36.6%), linalool (11.5–12.38%), and α-terpineol (6.7–7.0%) [96]. The second profile from Northeast Brazil was rich in compounds with *p*-methane and germacrane skeletons, as 1,8-cineole (63.3%), *p*-cymene (14.1%), α-terpinyl acetate (7.2%), and β-eudesmol (8.8%) [83,97]. The third profile also from Northeast Brazil showed *p*-menthane, cadinane and acyclic skeleton types, as *p*-cymene (5.1–17.8%), terpinolene (6.9–17.0%), γ-terpinene (10.3–17.1%), *epi*-α-cadinol (10.4–12.8%), linalool (4.7–7.3%), and δ-cadinene (3.8–5.3%) with a change of its constituents according to the seasons [25]. The fourth profile from Cuba was dominated by caryophyllane, bisabolane, germacrane, and pinane skeleton types, as caryophyllene oxide (39.8%), *ar*-turmerone (17.3%), β-gurjunene (6.7%), β-selinene (6.0%), and α-pinene (5.6%) [98].

### 4.9. Psidium sartorianum (O.Berg) Nied

It is native to Mexico, known as ‘arrayan’. Also, it occurs in Southeast Brazil, but there are reports of isolated occurrences in other regions [30].

Two *P. sartorianum* leaves oil records from Cuba and Mexico showed differences in their chemical compositions. The Cuba oil showed *p*-menthane (limonene 43.0%) and pinane (α-pinene 39.5% and β-pinene 5.6%) skeleton types [99]. The Mexico oil was dominated by the skeleton types of pinane (α-pinene 16.7%), caryophyllane (*E*-caryophyllene 12.4%), *p*-menthane (α-phellandrene 9.8%), and acyclic (*Z*-nerolidol 5.2%) [37].

### 4.10. Psidium striatulum DC

*Psidium striatulum* is a native and non-endemic species from Brazil, with occurrence in North (Pará, Roraima and Rondônia states), Northeast (Maranhão state), Midwest (Mato Grosso do Sul and Mato Grosso states), and South (Paraná state) regions. It is known as ‘araçá-mirim’ and ‘araçari’ in Roraima state, Brazil [100].

The fruits essential oil of *Psidium striatulum* from Roraima state, Brazil, showed constituents with pinane, caryophyllane, cadinane, and aromadendrene skeletons, as α-pinene (12%), α-humulene (10.4%), α-copaene (7.1%), globulol (5.7%), and aromadendrene (5.1%) [100]. Also, the leaf oil of another specimen existing in Maranhão state was rich in caryophyllane and germacrane skeletons, as *E*-caryophyllene (28.6%), α-selinene (7.7%), caryophyllene oxide (7.6%), β-selinene (7.4%), and selin-11-en-4-α-ol (6.0%) [56].

### 4.11. Other Species

Although 10 *Psidium* species have been widely studied with two or more essential oils or volatile concentrates samples, about 8 species were registered concerning their leaf oil compositions. These records were from Brazil and Cuba, with four oils in each country. The species that included only monoterpene constituents were *P. acutangulum* DC. with α-pinene 14.8%, 1,8-cineole 12.9%, β-pinene 10.1% [56]; *P. montanum* Sw. (syn. *Psidium wrightii* Krub et Urb.) with 1,8-cineole 46.9%, α-terpineol 9.2%, α-pinene 8.9% [101]; and *P. rotundatum* Griseb. with 1,8-cineole 28.0%, α-pinene 18.3%, α-terpineol 9.2%, *E*-nerolidol 8.7%, and linalool 5.1% [102]. Also, the species that included mono- and sesquiterpenes as major constituents were *P. brownianum* Mart. ex DC. with β-eudesmol 27.1%, 1,8-cineole 24.7%, α-elemol 11.8%, α-pinene 11.4%, guaiol 9.1%, and β-pinene 8.4% [103]; *P. cymosum* Urb. with *epi*-α-cadinol 46.6%, 1,8-cineole 15.0%, α-muurolol 11.8%, α-terpineol 8.4%, and α-pinene, 5.7% [99]; *P. gaudichaudianum* Proença & Faria with *E*-caryophyllene 17.0%, limonene 16.2%, α-pinene 8.4%, caryophyllene oxide 7.5%, and α-humulene, 5.8% [22]; *P. parvifolium* Griseb. with viridiflorol 31.9%, α-terpineol 8.2%, cubenol 7.3%, borneol 7.2%, *epi*-α-muurolol 6.6%, *trans*-sabinol 5.5% and *P. rufum* DC. (syn. *Psidium widgrenianum* O.Berg) with *E*-caryophyllene 21.0%, α-pinene 14.0%, γ-eudesmol 8.5%, 1,8-cineole 8.4%, α-eudesmol, 8.2%, and β-eudesmol, 6.8% [104].

## 5. Seasonal Variation in the Essential Oils Composition

**Figure 5:**
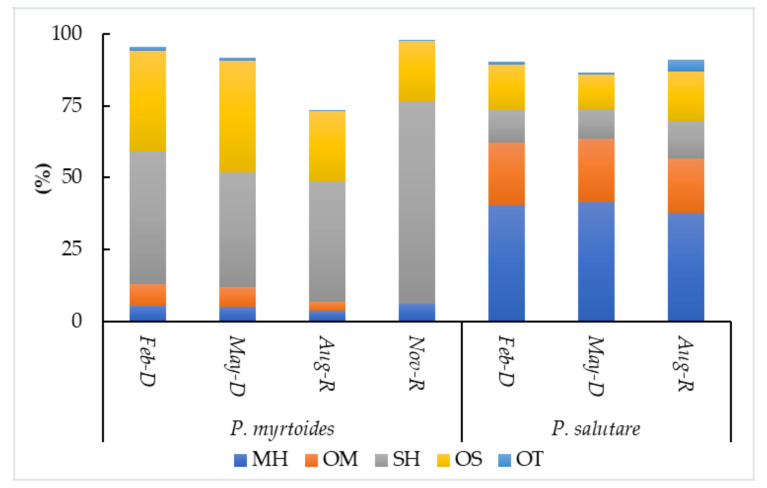
Chemical variability on essential oils of *Psidium myrtoides* and *P. salutare* during dry and rainy seasons. D: the dried season; R: the rainy season; MH: monoterpene hydrocarbons; OM: oxygenated monoterpenes; SH: sesquiterpene hydrocarbons; OS: oxygenated sesquiterpenes; OT: others.

Reports of *Psidium myrtoides* and *P. salutare* have focused on the effects of seasonality in the essential oils’ chemical composition. Both species were registered to the Chapada do Araripe, Ceará state, Brazil, with their leaves sampled in February, May, August, and November, which encompasses the dry and rainy seasons of that region [25,93]. The variation in the leaf essential oils is illustrated in Figure 5.

*Psidium myrtoides* oil presented the highest yields in the dry season (0.96–1.02%), decreasing in the rainy seasons (0.36–0.48%). A negative correlation between the oil yield and rainfall was noted. November presented the higher oil content (0.27 mL/100 g, fresh leaves), suggesting an excellent harvesting time. The oxygenated constituents (mono- and sesquiterpenes) decreased from May (OM: 7.01%; OS: 39.0%) to November (OM: 0%; OS: 21.0%), while the sesquiterpene hydrocarbons increased at the same time (May: 40.0%; November: 70.5%). The most predominant constituents in these two seasons were 1,8-cineole (29.5–48.1%), α-eudesmol (11.7–20.0%), α-pinene (5.0–12.8%), elemol (3.3–6.7%), and γ-eudesmol (2.5–5.8%). Lower content to 1,8-cineole occurred in March, during the ripening process of *Psidium* species [93].

The rainy season was considered the ideal period for the oil extraction of *Psidium salutare*, which showed the highest yield in February (0.73%), and decreasing in May (0.29%) and August (0.15%). The oil main constituents were *p*-cymene (5.1–17.8%), terpinolene (6.9–17.0%), γ-terpinene (10.3–17.1%), *epi*-α-cadinol (10.4–12.8%), linalool (4.7–7.3%), and δ-cadinene (3.8–5.3%). The results of seasonal studies do not show a statistical correlation with the environmental parameters [25].

## 6. Biological Activities

**Figure 6:**
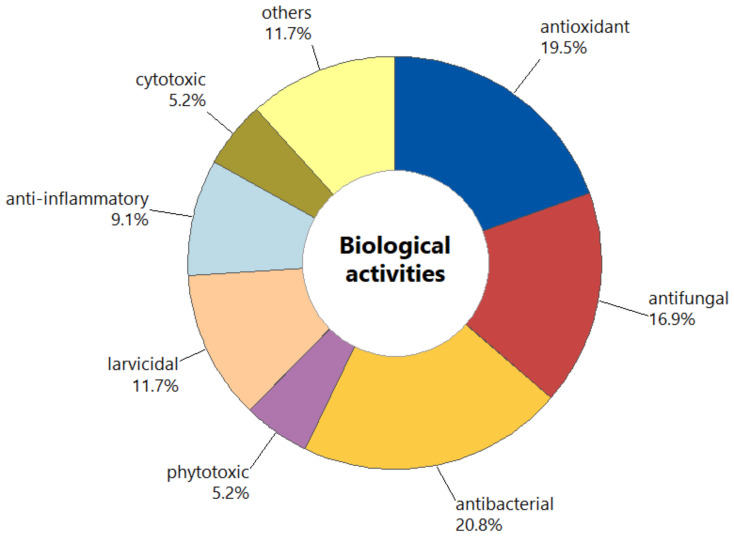
Distribution of records on biological activity of *Psidium* species.

The studies focused on *Psidium* essential oils’ biological activities comprised 77 records, which displayed at least one biological propriety. The percentage of biological assays, according to the number of studies, is presented in Figure 6. Antibacterial potential (20.8%) was the most investigated, followed by antioxidant (19.5%) and antifungal (16.5%). Also, other bioactivities have been reported, such as larvicide (11.7%), anti-inflammatory (11.7%), phytotoxic (5.2%), and cytotoxic (5.2%). Studies regarding antinociceptive, insecticide, nematicide, acaricide, vasorelaxant, spasmolytic, and anticholinesterase potential corresponded to approximately 11.7%.

### 6.1. Antioxidant Activity

Antioxidants compounds can prevent disorders and diseases caused by free radicals, stabilizing them. In recent years, the interest in replacing synthetic antioxidants with aromatic and medicinal plants has been growing. The following methods have evaluated the antioxidant activity of *Psidium* essential oils, DPPH, ABTS, linoleic acid oxidation, XO, OH, NO, ORAC, FRAP, deoxyribose degradation, and MDA [105].

The essential oil of *P. cattleyanum* showed a significant antioxidant potential by the DPPH method on TLC. The sesquiterpenes *E*-caryophyllene (22.5%), *neo*-intermedeol (14.2%), and β-selinene (10.1%) were identified as their main constituents [41]. On the other hand, the leaves oil rich in *E*-caryophyllene (59.6%), caryophyllene oxide (18.2%), and Z-caryophyllene (6.4%) was inactive on DPPH and ABTS methods (at 10–500 mg/mL), but it exhibited activity on linoleic acid oxidation assay (IC_50_ 56.41 μg/mL) [38].

*Psidium guajava* essential oil has been extensively investigated for its antioxidant potential, using different methods. The leaves oil from central region of Mauritius, dominated by caryophyllene oxide (15.4%) and limonene (11.6%), displayed antioxidant activity against DPPH (IC_50_ 5.19 µg/mL), ABTS (IC_50_ 3.09 µg/mL), XO (IC_50_ 2.51 µg/mL), OH (IC_50_ 1.90 µg/mL), NO (IC_50_ 2.71 µg/mL), ORAC (0.27 gTE/gEO), and FRAP (44.41 µmol Fe^+2^/mg OE) methods [62]. *Psidium guajava* essential oils from different China regions were evaluated by DPPH, ABTS, and FRAP methods. Three oil records rich in oxygenated sesquiterpenes (17.4–18.7%) from Guangdong province showed the highest antioxidant potential on DPPH (IC_50_ 18.5–20.3 mg/mL), ABTS (13.1–16.2 mg/mL) and FRAP assays (7.3–9.1 mmol Vc/g DM). On the other hand, the samples from other regions were less active on DPPH (IC_50_ 20.4–33.7 mg/mL) and ABTS (IC_50_ 18.3–25.3 mg/mL) methods [53]. *Psidium guajava* leaves (*E*-caryophyllene 16.9%, selin-7(11)-en-4-α-ol 8.3%, and α-selinene 6.5%) and fruits (*E*-caryophyllene 17.6%, limonene 11.0%, and α-selinene, 6.6%) oils from Egypt displayed antioxidant activity on DPPH (leaves oil, IC_50_ 3,59 mg/mL; fruits oil, IC_50_ 8,11 mg/mL), and deoxyribose assay (leaves oil, IC_50_ 12,64 μg/mL; fruits oil, IC_50_ 42,78 μg/mL). This antioxidant effect may result from the high content of oxygenated mono- and sesquiterpenes in both oils [57].

*Psidium guineense* essential oil collected in Brazil and rich in spathulenol (80.7%) showed high antioxidant activity on DPPH (IC_50_ 63.1 µg/mL), ABTS (IC_50_ 780.1 µg/mL), and MDA (IC_50_ 37.9 µg/mL) methods. This effect may be attributed to spathulenol, its principal constituent [84].

### 6.2. Antifungal Activity 

The growing resistance to azole antimycotics, the most used antifungals class, has been a recurrent problem in the treatment of fungal pathologies. Today new efforts are dedicated to discovering new antimycotics agents with different mechanisms of action [106]. Therefore, the use of natural products stands out as a viable alternative for the treatment of several mycoses due to its broad spectrum of bioactive compounds [107]. In this context, essential oils gain prominence as fungicides, with many studies addressing this aspect [108].

*Psidium cattleyanum* essential oil—rich in *E*-caryophyllene (59.6%), caryophyllene oxide (18.2%), and *Z*-caryophyllene (6.4%)—was evaluated against *Candida* spp. fungi known to cause superficial and invasive infections to debilitated or immunocompromised patients. Its oil did not show activity against *Candida lipolytica* (MIC 125.0 mg/mL), *C. parapsilosis* (MIC 104.2 mg/mL), *C. guilhermondi* (MIC 125.0 mg/mL), *C. albicans* (MIC 166.7 mg/mL), and *Trichosporon asahii* (MIC 41.67 mg/mL) [38].

*Psidium guajava* oil, rich in limonene (33.3%), α-pinene (29.5%), and carvotacetone acetate (8.2%), was evaluated against *Candida albicans* (MIC 0.14 mg/mL), *Rhodotorula glutinis* (MIC 0.09 mg/mL), *Schizosaccharomyces pombe* (MIC 0.09 mg/mL), *Saccharomyces cerevisiae* (MIC 0.06 mg/mL), *Yarrowia lypolitica*, (MIC 0.23 mg/mL), displaying significant activity [72]. Furthermore, *Psidium guajava* oil records from different regions of China, rich in *E*-caryophyllene (27.2–31.4%), exhibited activity against *Saccharomyces cerevisiae* (inhibition halo 16.9–20.6 mm) and *Rhodotorula* sp. (inhibition halo 18.3–26.3 mm) [53].

*Psidium myrtoides* oils, rich in 1,8-cineole (29.5–48.1%), α-eudesmol (11,7–20.0%), α-pinene (5.0–12.8%), elemol (3.3–6.7%), and γ-eudesmol (2.5–5.8%), with seasonal influence on its chemical compositions, showed significant fungicidal activity against *Candida albicans* (MFC 1.0–4.1 µg/mL, IC_50_ 103.3–963.8 µg/mL), and moderate against *C. krusei* (MFC 8.2–16.4 µg/mL, IC_50_ 1235.9–3564.5 µg/mL) and *C. tropicalis* (MFC > 16.384 µg/mL, IC_50_ 1671.1–2535.1 µg/mL) [93].

*Psidium salutare* oil, rich in *p*-cymene (5.1–17.8%), terpinolene (6.9–17.0%), γ-terpinene (10.3–17.1%), *epi*-α-cadinol (10.4–12.8%), linalool (4.7–7.3%), and δ-cadinene (3.8–5.3%) exhibited significant activity against *Candida albicans* (MFC 1.0–4.1 µg/mL), *Candida krusei*, (MFC 8.2–16.4 µg/mL), and *Candida tropicalis* (MFC > 16.4 mg/mL) [25].

The fungicidal activity of *these Psidium* species can be attributed to the mono- and sesquiterpenes present in their essential oils, as these compound classes showed fungicidal potential previously reported [109,110].

### 6.3. Antibacterial Activity

In the last years, the antimicrobial resistance to antibiotics has increased due to the adaptive evolution of bacteria and fungi. Thus, the treatment of pathologies caused by these agents has been hampered. For this reason, research focused on the potential of new antimicrobials based on natural products has been explored, especially essential oils, generating an excellent source of bioactive compounds for the pharmaceutical industry, among others [111,112,113].

Several published works have reported the potential of *Psidium* essential oils against Gram-positive and Gram-negative bacteria. *Psidium guajava* essential oils from China were evaluated by disc diffusion method exhibiting inhibition halos against strains of *Bacillus aryabhattai* (15.3–23.1 mm), *Arthrobacter creatinolyticus* (9.1–20.1 mm), *Bacillus megaterium* (16.9–21.2 mm), *Bacillus subtilis* (17.0–19.0 mm) [53], *Enterococcus faecales* (6.0–16.5 mm), *Staphylococcus aureus* (9.0–18.6 mm), *Haemophilus influenzae* (12.0 mm), *Pseudomonas aeruginosa* (6.0–8.0 mm), and *Escherichia coli* (13.0–19.4 mm), methicillin-resistant *Staphylococcus aureus* (7.6 mm), and *Staphylococcus epidermidis* (18.2 mm) [71,73].

*Psidium cattleyanum* essential oils showed significant antibacterial activity against *Porphyromonas gingivalis* (MIC 20.0 µg/mL), *Prevotella nigrescens* (MIC 62.5 µg/mL), *Fusobacterium nucleatum* (MIC 12.5 µg/mL), *Bacteroides fragilis* (MIC 12.5 µg/mL), *Actinomyces naeslundii* (MIC 50 µg/mL), *Aggregatibacter actinomycetemcomitans* (MIC 6.2 µg/mL), *Peptostreptococcus anaerobius* (MIC 62.5 µg/mL [44], and *Neisseria gonorrhoeae* (MIC 13.0 µg/mL) [40].

*Psidium guineense* essential oil displayed effective antibacterial activity against *Mycobacterium tuberculosis* (MIC 126.4 µg/mL) [84]. The same with *P. myrtoides* oils against *Streptococcus* strains, as *S. mitis* (MIC 100 µg/mL), *S. sanguinis* (MIC 100 µg/mL), *S. sobrinus* (MIC 250 µg/mL), *S. salivarius* (MIC 250 µg/mL), and *S. mutans* (MIC 62.5 µg/mL) [91].

### 6.4. Phytotoxic Activity

The phytotoxic potential of plants and their chemical constituents against invasive plants is increasingly investigated as a possible alternative to synthetic herbicides in crops weed control [114]. Some *Psidium* essential oils inhibited parasitic herbs’ growth, showing the potential to be used against the crops’ invasive plants [22].

Leaf essential oils of four *Psidium* species from Espírito Santo state, Southeast Brazil, were evaluated against *Lactuca sativa* L. and *Sorghum bicolor* (L.) Moench. Germination inhibition and germination speed index were the analyzed parameters. *Psidium cattleyanum* oil, rich in *E*-caryophyllene (23.4%), caryophyllene oxide (11.5%), and α-pinene (11.3%), at a concentration of 3000 µg/mL, caused germination inhibition against *L. sativa* (74.6%) and *S. bicolor* (92.6%), with a germination speed index of 3.4 mm and 6.9 mm, respectively. *Psidium friedrichsthalianum* oil dominated by *E*-caryophyllene (24.6%), caryophyllene oxide (10.6%), α-humulene (9.2%), and α-copaene (5.9%), at a concentration of 375 µg/mL, inhibited the germination of *L. sativa* (92.8%) and *S. bicolor* (91.7%), with a germination speed index of 5.7 mm and 8.4 mm, respectively. *Psidium gaudichaudianum*, rich in *E*-caryophyllene (17.0%), limonene (16.2%), α-pinene (8.4%), caryophyllene oxide (7.5%), and α-humulene (5.8%), at a concentration of 1500 µg/mL, caused a germination inhibition of *L. sativa* (90.7%) and *S. bicolor* (91.1%), with a germination speed index of 5.2 mm and 8.1 mm, respectively. Also, *P. myrtoides* oil, composed primarily of *E*-caryophyllene (19.4%), α-bisabolol (10.4%), α-humulene (10.4%), α-copaene (6.3%), and caryophyllene oxide (5.3%), at a concentration of 3000 µg/mL, inhibited the germination of *L. sativa* and *S. bicolor* in 47.4% and 90.4%, at a germination speed index of 3.4 mm and 7.8 mm, respectively [22].

### 6.5. Larvicidal Activity

The larvicidal effect of essential oils has been tested against several disease vectors [115]. Thus, the *P. guajava* essential oil from Nepal—rich in *E*-nerolidol (35.6%), *E*-caryophyllene (15.8%), and 2*Z*,6*E*-farnesol (6.7%)—exhibited significant larvicidal activity against *Chaoborus plumicornis* (LC_50_ 63.3 μg/mL) [51]. Moreover, *Psidium guajava* oil from Espirito Santo state, Brazil, dominated by *E*-caryophyllene (26.6%), caryophyllene oxide (15.5%), and aromadendrene epoxide (8.1%), showed larvicidal activity against *Aedes aegypti* larvae (LC_50_ 39.48–64.25 μg/mL) [55]. Likewise, *P. myrtoides* oils from Maranhão state, Brazil, rich in *E*-caryophyllene (26.1–31.0%), α-humulene (12.3–23.9%), and caryophyllene oxide (7.3–10.1%) showed significant activity against *Artemia salina* (LC_50_ 95.3 µg/mL) and low activity on *Aedes aegypti* larvae (LC_50_ 292 mg/mL) [89].

### 6.6. Anti-Inflammatory

Natural products with anti-inflammatory activity have long been used in folk medicine for inflammatory diseases and their symptoms, such as fever, pain, migraine, and arthritis [116]. Scientists are focused on herbal medicine research and active compounds to develop new drugs as useful therapeutic agents [117]. Thus, essential oils gain importance in the field of human health, acting as anti-inflammatories [118].

Three *P. guajava* leaf oils recorded in Rio de Janeiro, Brazil, showed anti-inflammatory activity in pleurisy induced by lipopolysaccharide model with inhibition in migration of eosinophils between 67% and 76% at 100 mg/kg. In these oils, predominated the caryophyllane-type compounds, as α-humulene (13.0–37.0%), *E*-caryophyllene (7.2–24.0%), β-selinene (7.7–14.0%), α-selinene (10.0–12.0%), α-muurolol (7.6–9.6%), cedr-8-(15)-en-9-α-ol (7.4–7.6%), humulene epoxide II (6.6%), and caryophyllene oxide (5.0%) [67]. Also, leaf and fruit essential oils of *P. guajava* from Egypt, rich in *E*-caryophyllene (16.9% and 17.6%), limonene (0.2% and 11.0%), selin-7(11)-en-4-α-ol (8.3% and 0.5%), α-selinene (6.5% and 6.6%), β-selinene (6.3% and 6.4%), 1,8-cineole (5.4% and 0.8%), and δ-cadinene (5.3% and 4.9%), showed a moderate activity when tested in vitro on 5-lipoxygenase inhibition, exhibiting IC_50_ values of 49.76 μg/mL for the fruits, and 32.53 μg/mL for the leaves [57].

*Psidium guineense* leaf oil from Mato Grosso do Sul, Brazil, dominated by spathulenol (80.1%) showed the inhibition of 59.46% in a carrageenan-induced mouse paw model, at 300 mg/kg, reduction of 45.33% (at 30 mg/kg) and 77.70% (at 100 mg/kg) in the increase of total leukocytes in pleurisy model, and reduction in the rise of protein levels of 49.72% (at 30 mg/kg) and 78.40% (at 100 mg/kg) [84].

The leaf oil of *Psidium rufum* from Rio de Janeiro, Brazil, composed primarily by *E*-caryophyllene (21.0%), α-pinene (14.0%), γ-eudesmol (8.5%), 1,8-cineole (8.4%), α-eudesmol (8.2%), and β-eudesmol (6.8%), showed 70% reduction (at 100 mg/kg) in eosinophil migration in Zymosan assay, and 51% reduction (at 100 mg/kg) in nitric oxide production, in vitro [104].

### 6.7. Cytotoxic

The anticancer potential of essential oils has been investigated, aiming to implement them as therapeutic agents, whether in alternative or complementary treatment [119]. The cytotoxic activity of essential oils from *P. guajava*, *P. guineense,* and *P. myrtoides* were evaluated against the human cancer cell lines. Oils of leaves and fruits of *P. guajava* from Egypt, dominated by *E*-caryophyllene (16.9% and 17.6%), limonene (0% and 11.0%), and selin-7(11)-en-4α-ol (8.3% and 0%), respectively, showed low activity against hepatic cancer cell lines (HepG2) (leaf oil, IC_50_ 130.69 µg/mL; fruit oil, IC_50_ 196.45 µg/mL), breast cancer (MCF-7) (leaf oil, IC_50_ 351.00 µg/mL; fruit oil, IC_50_ 544.38 µg/mL) [57]. Oils of leaves of *P. myrtoides* from Brazil, rich in *E*-caryophyllene (30.9%), α-humulene (15.9%), and α-copaene (7.8%), displayed low cytotoxic potential against the breast adenocarcinoma (MCF-7, IC_50_ 254.5 μg/mL) and cervical adenocarcinoma (HeLa, IC_50_ 324.2 μg/mL) human cells [91]. *Psidium guineense* oil, rich in spathulenol, (80.7%), showed high cytotoxic activity against the human cells: ovarian expressing the phenotype of multiple drug resistance (NCI-ADR/RES, IC_50_ 9.25 µg/mL), renal (786–0, GI_50_ 2.57 µg/mL), lung (NCI-H460, GI_50_ 4.57 µg/mL), prostate (PCO-3, GI_50_ 9.18 µg/mL), ovarian (OVCAR-3, GI_50_ 0.89 µg/mL), colon (HT-29, GI_50_ 5.62 µg/mL), leukemia (K-562, GI_50_ 5.03 µg/mL), keratinocytes (HaCaT, GI_50_ 7.98 µg/mL), glioma (U251, GI_50_ 9.84 µg/mL), and breast (MCF-7, GI_50_ 7.90 µg/mL) [84].

### 6.8. Other Activity

Other biological activities concerning *Psidium* essential oils. *Psidium pohlianum* O. Berg (syn. *Psidium salutare* var. *pohlianum* (O. Berg) Landrum), rich in β-eudesmol (27.1%), 1,8-cineole (24.7%), α-elemol (11.8%), α-pinene (11.4%), guaiol (9.1%), and β-pinene (8.4%), showed an antinociceptive effect in mice, causing a reduction in nociception induced by formalin of 25% (dose of 100 mg/kg), 45% (dose of 200 mg/kg), and 84% (dose of 400 mg/kg), respectively. In the hot plate test, this oil increased the reaction times by 30 min (200 mg/kg) and 60 min (400 mg/kg), respectively. In the tail compression and locomotion tests, positive results were also observed [97]. Moreover, in the oil of *Psidium brownianum* Mart. ex DC., rich in β-eudesmol (27.1%), 1,8-cineole (24.7%), α-elemol (11.8%), α-pinene (11.4%), guaiol (9.1%), and β-pinene (8.4%), the antinociceptive effect has also been observed in the acute nociception model, induced by acetic acid at 100 and 200 mg/kg doses, where the number of abdominal contortions was reduced by 41.79%. This oil reduced paw licking time in both phases, in formalin-induced abdominal contortions, by 80.47% (100 mg/kg) and 87.59% (200 mg/kg), respectively. In the hot plate test, at a concentration of 100 and 200 mg/kg, the oil prolonged the mice’s reaction time (30 min, 68.54, and 76.16%; 60 min, 105.47, and 106.52%; 90 min, 105.57, and 96.58%, respectively). In capsaicin-induced nociception, the oil reduced the animals’ paw licking time by 45.36% and 42.26% (doses of 100 and 200 mg/kg, orally administered) after capsaicin (20 μL/paw) administration [103].

Thr leaf oil of *Psidium guajava* shown promise as natural insecticides on the fumigation method against *Tribolium castaneum* (LC_50_ 6.1, <2 µg/L of air after 24 and 72 h treatment), *Culex pipiens* (LC_50_ > 50 µg/L) [70] and *Drosophila melanogaster* (LC_50_ 327 μg/mL) [51]. Also, *Psidium guajava* oil showed nematicidal activity against *Caenorhabditis elegans* (LC_50_ 142 μg/mL) [51] and acaricidal against *Rhipicephalus microplus* females on the adult immersion test, showing great efficacy (99.9%) at 12.5 mg/mL [58]. The fruit oil of *Psidium guajava*, rich in hexanol (13.9%), cinnamyl alcohol (10.9%), and butanol (10.7%) displayed vasorelaxant activity inhibiting the K^+^ and phenylephrine with EC_50_ of 5.52 and 6.23 mg/mL, respectively, besides spasmodic effects inhibiting the spontaneous and induced K^+^ contractions with EC_50_ of 0.84 and 0.71 mg/mL, respectively [75].

*Psidium cattleyanum* oil, rich in *E*-caryophyllene (59.6%), caryophyllene oxide (18.2%), and *Z*-caryophyllene (6.4%), showed low toxicity when tested in a mouse model, with oral administration (LD_50_ > 500 mg/Kg) [38]. Also, *P. salutare* oil, rich in α-pinene (12%), α-humulene (10.4%), α-copaene (7.1%), globulol (5.7%), and aromadendrene (5.1%), presented moderate inhibition for the acetylcholinesterase enzyme (44.4%) [99].

## 7. Conclusions

The *Psidium* genus essential oils present a significant chemical variability. They are composed of mono- and sesquiterpenes with acyclic (C_10_ and C_15_), *p*-menthane, pinane, bisabolane, germacrane, caryophyllane, cadinane, and aromadendrene skeleton-types. Geographical occurrence and seasonality can influence the chemical composition of *Psidium* essential oils. Also, it has exhibited a wide range of biological activities, directly influenced by its chemical variability. Although *Psidium* species display a broad spectrum of ethnomedicinal uses, studies on their biological activities are mostly restricted to *P. guajava* and *P. cattleyanum* species. Furthermore, a reduced number of species with known chemical composition, 18 species of 266 contained in the genus, from 110 studied samples. Thus, it is necessary to further explore the volatile content of *Psidium* species and their therapeutic properties. Variations in the chemical profile of the species indicate the importance of optimizing protocols for the collection, processing, and extraction of plant material.

## Figures and Tables

**Figure A1:**
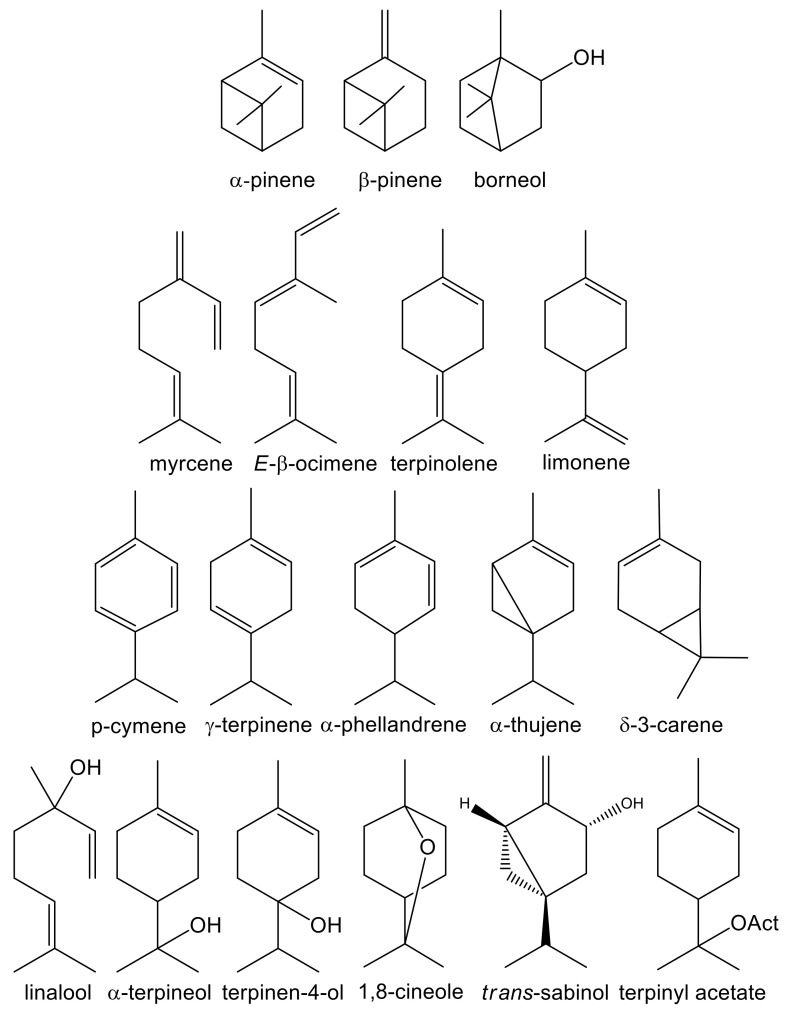
Major monoterpenoids found in *Psidium* species.

**Figure A2:**
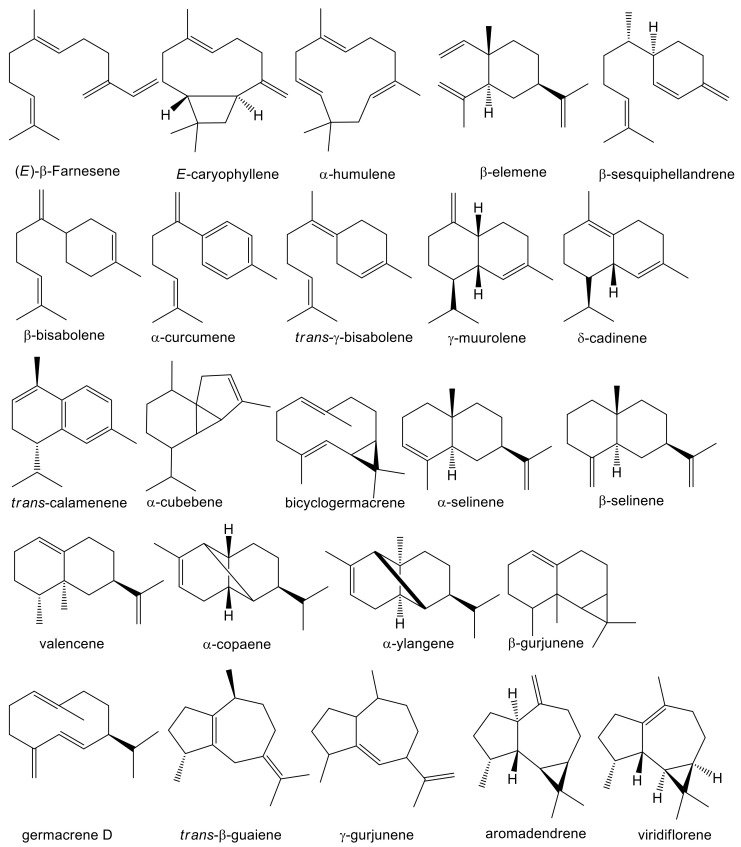
Major sesquiterpene hydrocarbons found in *Psidium* species.

**Figure A3:**
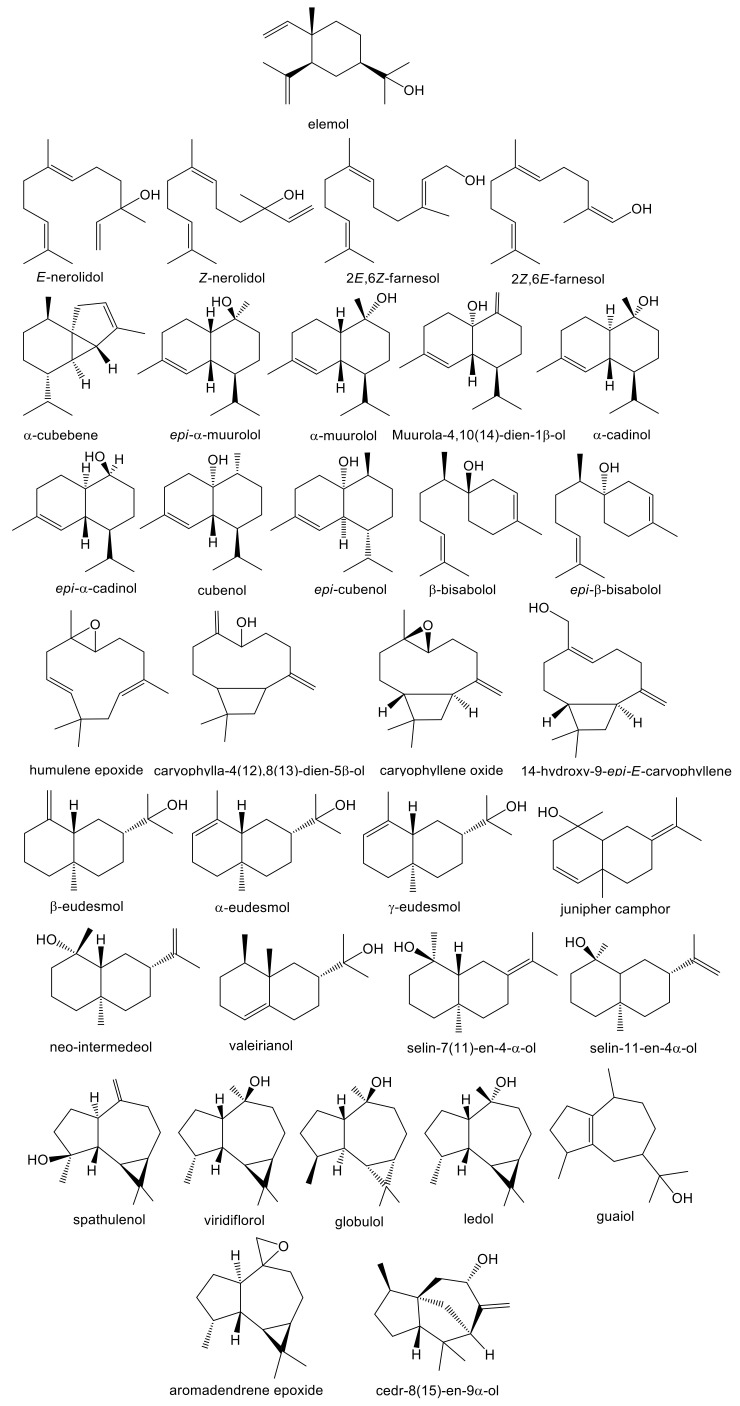
Major oxygenated sesquiterpenes found in *Psidium* species.

**Figure A4:**
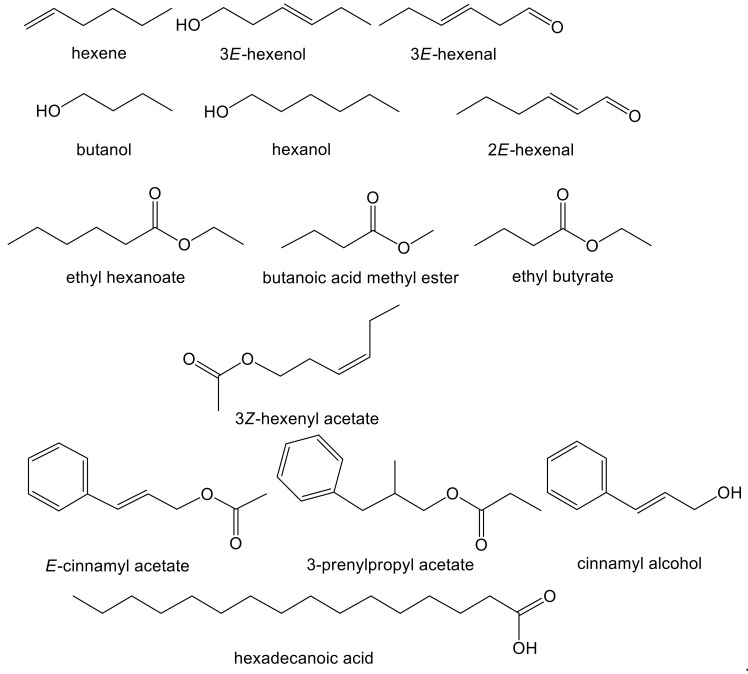
Other Major compound found in *Psidium* species.

## Data Availability

The data presented in this study are available in this article.

## Abbreviations

DPPH	2,2-diphenyl-1-picrylhydrazyl
EO	Essential oil
FRAP	Ferric reducing antioxidant power
HD	Hydrodistillation
HS-SPME	HeadSpace Solid Phase Micro Extraction,
IC_50_	Median inhibitory concentration
LC_50_	Median lethal concentration
MDA	Malondialdehyde
MFC	Minimum Fungicide Concentration
MIC	Minimum inhibitory concentration
MMC	Minimum microbicide concentration
NO	Nitric oxide radical scavenging assay
ORAC	Oxygen radical absorbing capacity assay
SAFE	Solvent-assisted flavor evaporation
SD	Steam distillation
SDE	Simultaneous steam distillation-solvent extraction,
SE	Solvent extraction
SPME	Solid Phase Micro Extraction
TBARS	Thiobarbituric Acid Reactive Species
TLC	Thin Layer Chromatography
XO	Xanthine oxidase assay
TE/gEO	Trolox equivalent per gram of essential oil
µmol Fe^+2^/mg OE	Micromol of FE^+2^ per milligram of essential oil

## Appendix A

**Table A1:** *Psidium* species: essential oil composition

Species	Occurrence	Plant Part/ Extraction Type	Primary Components (>5%)	Ref.
*P. acutangulum*	Boa vista-Bonfim Road, Roraima, Brazil	Leaf/stems (HD)	α-pinene (14.8%), 1,8-cineole (12.9%), and β-pinene (10.1%)	[56]
*P. brownianum*	Crato, CE, Brazil	Leaf (HD)	β-eudesmol (27.1%), 1,8-cineole (24.7%), α-elemol (11.8%), α-pinene (11.4%), guaiol (9.1%), and β-pinene (8.4%)	[103]
*P. cattleyanum*	Moorea Island, Haapiti, French Polynesia	Leaf (HD)	Profile I (caryophyllane): *E*-caryophyllene (31.5%)	[36]
*P. cattleyanum*	Arizona, USA	Leaf (SD)	Profile II (caryophyllane): *E*-caryophyllene (59.9%) and caryophyllene oxide (5.4%)	[37]
*P. cattleyanum*	Pelotas, RS, Brazil	Leaf (SD)	Profile III (caryophyllane): *E*-caryophyllene (59.6%), caryophyllene oxide (18.2%), and Z-caryophyllene (6.4%)	[38]
*P. cattleyanum*	Honolulu, Hawaii, USA	Leaf (HD)	Profile IV (caryophyllane/pinane/acyclic): *E*-caryophyllene (59.0%), α-pinene (13.2%), and myrcene (11.3%)	[39]
*P. cattleyanum*	Alegre, ES, Brazil	Leaf (HD)	Profile V (caryophyllane/pinane): *E*-caryophyllene (23.4%), caryophyllene oxide (11.5%), and α-pinene (11.3%)	[22]
*P. cattleyanum*	El-Behera, Egypt	Leaf (HD)	Profile VI (caryophyllane/pinane/acyclic)*: E*-caryophyllene (28.8%), α-pinene (28.0%), myrcene (13.4%), and *trans*-β-ocimene (5.3%)	[40]
*P. cattleyanum*	Pelotas, RS, Brazil	Fruit (HD)	Profile VII (caryophyllane/eudesmane/aromadendrene)*: E*-caryophyllene (22.5%), *neo*-intermedeol (14.2%), β-selinene (10.1%), *trans*-β-guaiene (9.1%), and α-humulene (7.5%)	[41]
*P. cattleyanum*	Pinar del Río, Cuba	Leaf (HD)	Profile VIII (cadinane/caryophyllane)*: epi*-α-muurolol (21.9%), α-cadinol (20.0%), *epi*-α-cadinol (16.7%), caryophyllene oxide (13.6%), juniper camphor (9.4%), and 14-hydroxy-9-*epi*-*E*-caryophyllene (5.7%)	[42]
*P. cattleyanum*	Atlantic Forest, Brazil	Leaf (HD)	Profile IX (*p*-menthane/caryophyllane/eremophilane/acyclic): α-thujene (25.2%), 1,8-cineole (16.4%), *E*-caryophyllene (10.2%), valencene (8.0%), and myrcene (5.0%)	[43]
*P. cattleyanum*	Limeira, SP, Brazil	Leaf (HD)	Profile X (eudesmane/caryophyllane/*p*-menthane/aromadendrene) viridiflorol (17.9%), *E*-caryophyllene (11.8%), 1,8-cineole (10.8%), β-selinene (8.6%), α-humulene (6.0%), and aromadendrene (5.0%)	[44]
*P. cymosum*	Pinar del Rio, Cuba	Leaf (HD)	*epi*-α-cadinol (46.6%), 1,8-cineole (15.0%), α-muurolol (11.8%), α-terpineol (8.4%), and α-pinene (5.7%)	[99]
*P. friedrichsthalianum*	San Jose, Costa Rica	Leaf (SD)	Profile I (caryophyllane/elemane/pinane/germacrane/cadinane): *E*-caryophyllene (36.8%), β-elemene (12.86%), α-pinene (10.6%), bicyclogermacrene (8.3%), β-pinene (8.3%), and α-ylangene (7.8%)	[37]
*P. friedrichsthalianum*	Alegre, ES, Brazil	Leaf (HD)	Profile II (caryophyllane/cadinane): *E*-caryophyllene (24.6%), caryophyllene oxide (10.6%), α-humulene (9.2%), and α-copaene (5.9%)	[22]
*P. gaudichaudianum*	Alegre, ES, Brazil	Leaf (HD)	*E*-caryophyllene (17.0%), limonene (16.2%), α-pinene (8.4%), caryophyllene oxide (7.5%), and α-humulene (5.8%)	[22]
*P. guajava*	Saint-Denis, France	Fruit (HD)	Profile I (caryophyllane/acyclic/aromadendrene): *E*-caryophyllene (24.6%), nerolidol (18.0%), and caryophyllene oxide (5.1%)	[49]
*P. guajava*	Sindh Province, Pakistan	Leaf (HD)	Profile I (caryophyllane/acyclic/aromadendrene): *E*-caryophyllene (20.3%), globulol (8.2%), and *E*-nerolidol (7.7%)	[50]
*P. guajava*	Kathmandu, Nepal	Leaf (HD)	Profile I (caryophyllane/acyclic/aromadendrene): *E*-nerolidol (35.6%), *E*-caryophyllene (15.8%), (2*Z*,6*E*)-farnesol (6.7%), and ledol (5.5%)	[51]
*P. guajava*	Guira de Melena, Havana, Cuba	Leaf (HD)	Profile II (caryophyllane/acyclic/eudesmane/aromadendrene): *E*-caryophyllene (21.6%), *E*-nerolidol (l9.2%), selin-ll-en-4α-ol (13.4%), viridiflorene (8.8%), α-selinene (8.3%), caryophyllene oxide (8.2%), and cedr-8(15)-en-9α-ol (7.9%)	[52]
*P. guajava*	Shijiazhuang, Hebei, China	Leaf (HD)	Profile III (caryophyllane/aromadendrene): *E*-caryophyllene (27.4%) and γ-gurjunene (13.5%)	[53]
*P. guajava*	Anguo, Hebei, China	Leaf (HD)	Profile III (caryophyllane/aromadendrene): *E*-caryophyllene (24.4%) and γ-gurjunene (12.7%)	[53]
*P. guajava*	Hangzhou, Zhejiang, China	Leaf (HD)	Profile III (caryophyllane/aromadendrene): *E*-caryophyllene (31.4%) and γ-gurjunene (14.0%),	[53]
*P. guajava*	Chenchou, Tunisia	Leaf (HD)	Profile III (caryophyllane/aromadendrene): viridiflorol (36.4%) and *E*-caryophyllene (5.9%)	[54]
*P. guajava*	Panyu, Guangdong, China	Leaf (HD)	Profile IV (caryophyllane/cadinane/aromadendrene): *E*-caryophyllene (25.7%), calamenene (7.4%), γ-gurjunene (9.5%), and *epi*-α-cadinol (6.4%)	[53]
*P. guajava*	Meizhou, Guangdong, China	Leaf (HD)	Profile IV (caryophyllane/cadinane/aromadendrene): *E*-caryophyllene (25.0%), γ-gurjunene (9.5%), *epi*-α-cadinol (6.1%), and calamenene (7.8%)	[53]
*P. guajava*	Taipei, Taiwan	Leaf (HD)	Profile IV (caryophyllane/cadinane/aromadendrene): *E*-caryophyllene (17.2), γ-gurjunene (9.3%), *epi*-α-cadinol (10.0%), and calamenene (6.7%)	[53]
*P. guajava*	Tainan, Taiwan	Leaf (HD)	Profile IV (caryophyllane/cadinane/aromadendrene): *E*-caryophyllene (21.4%), γ-gurjunene (9.2%), *epi*-α-cadinol (7.8%), and calamenene (6.6%)	[53]
*P. guajava*	Jiangmen, Guangdong, China	Leaf (HD)	Profile IV (caryophyllane/cadinane/aromadendrene): *E*-caryophyllene (25.0%), calamenene (7.1%), γ-gurjunene (9.5%), and *epi*-α-cadinol (6.0%)	[53]
*P. guajava*	Alegre, ES, Brazil	Leaf (HD)	Profile V (caryophyllane/bisabolane/*p*-menthane/acyclic): *E*-caryophyllene (7.6%), β-bisabolol (19.5%), limonene (17.8%), 1,8-cineole (5.1%), and *E*-nerolidol (6.9%)	[55]
*P. guajava*	Alegre, ES, Brazil	Leaf (HD)	Profile V (caryophyllane/bisabolane/*p*-menthane/acyclic): *E*-caryophyllene (9.4%), β-bisabolol (15.1%), limonene (6.5%), α-humulene (16.5%), *E*-nerolidol (7.4%), β-bisabolene (6.3%), and humulene epoxide II (6.0%)	[55]
*P. guajava*	Belém, PA, Brazil	Leaf/stems (HD)	Profile V (caryophyllane/bisabolane/*p*-menthane/acyclic): α-pinene (23.9%), 1,8-cineole (21.4%), β-bisabolol (9.2%), *E*-caryophyllene (5.2%), and *E*-nerolidol (5.0%)	[56]
*P. guajava*	Bozhou, Anhui, China	Leaf (HD)	Profile VI (caryophyllane/bisabolane/aromadendrene): *E*-caryophyllene (26.4%), β-bisabolene (5.23%), and γ-gurjunene (15.2%)	[53]
*P. guajava*	Cairo, Egypt	Leaf (HD)	Profile VII (caryophyllane/eudesmane/*p*-menthane/cadinane): *E*-caryophyllene (16.9%), selin-7(11)-en-4-α-ol (8.3%), α-selinene (6.5%), β-selinene (6.3%), 1,8-cineole (5.4%), and δ-cadinene (5.3%)	[57]
*P. guajava*	Alegre, ES, Brazil	Leaf (HD)	Profile VIII (caryophyllane/eudesmane/aromadendrene): *E*-caryophyllene (26.6%), selin-11-en-4α-ol (6.7%), caryophyllene oxide (15.5%), aromadendrene epoxide (8.1%), β-selinene (7.6%), and α-selinene (6.5%)	[55]
*P. guajava*	Alegre, ES, Brazil	Leaf (HD)	Profile IX (caryophyllane/eudesmane/*p*-menthane/aromadendrene): *E*-caryophyllene (19.4%), selin-11-en-4α-ol (7.4%), caryophyllene oxide (16.6%), aromadendrene epoxide (9.2%), 1,8-cineole (8.4%), and β-selinene (5.6%)	[55]
*P. guajava*	Alegre, ES, Brazil	Leaf (HD)	Profile X (caryophyllane/eudesmane/*p*-menthane/aromadendrene/cadinane): *E*-caryophyllene (10.2%), selin-11-en-4α-ol (16.7%), caryophyllene oxide (8.0%), aromadendrene epoxide (9.5%), 1,8-cineole (7.6%), *epi*-α-cadinol (7.9%), β-selinene (8.2%), *epi*-cubenol (6.7%), and α-selinene (6.3%)	[55]
*P. guajava*	Moorea Island, Taravao, French Polynesia	Leaf (HD)	Profile X (caryophyllane/eudesmane/*p*-menthane/bisabolane/aromadendrene): *E*-caryophyllene (18.3%), selin-11-en 4α-ol (6.9%), 1,8-cineole (6.2%), and *E*-α-bisabolene (5.5%)	[36]
*P. guajava*	Parnaíba, PI, Brazil	Leaf (SD)	Profile X (caryophyllane/eudesmane/*p*-menthane/bisabolane): *E*-caryophyllene (39.0%), β-selinene (9.7%), α-selinene (9.7%), 1,8-cineole (6.9%), and aromadendrene (6.3%)	[58]
*P. guajava*	Cairo, Egypt	Fruit (SE)	Profile X (caryophyllane/eudesmane/*p*-menthane/bisabolane): *E*−caryophyllene (12.8%), α−selinene (8.4%), β−selinene (8.3%), and 3*Z*-hexenol (6.7%)	[59]
*P. guajava*	Chang-hua, Taiwan	Leaf (SD)	Profile XI (caryophyllane/aromadendrene/*p*-menthane): *E*-caryophyllene (27.7%), α-pinene (14.7%), 1,8-cineole (12.4%), and aromadendrene (6.6%)	[60]
*P. guajava*	Cairo, Egypt	Fruit (HD)	Profile XI (caryophyllane/eudesmane/*p*-menthane): *E*-caryophyllene (17.6%), limonene (11.0%), α-selinene (6.6%), and β-selinene (6.4%)	[57]
*P. guajava*	Guira de Melena, Havana, Cuba	Fruit (SDE)	Profile XI (caryophyllane/*p*-menthane/others): *E*-caryophyllene (12.2%), limonene (10.3%), hexadecanoic acid (8.7%), and 2*E*-hexenal (5.2%)	[61]
*P. guajava*	Mauritius	Leaf (HD)	Profile XI (caryophyllane/*p*-menthane): caryophyllene oxide (15.4%) and limonene (11.6%)	[62]
*P. guajava*	Lucknow, Uttar Pradesh, India	senescent leaves (HD)	Profile XI (caryophyllane/*p*-menthane): limonene (29.1%), *E*-caryophyllene (15.7%), caryophyllene oxide (8.8%), and caryophylla-4(12),8(13)-dien-5β-ol (6.5%)	[63]
*P. guajava*	Jeju Island, South Korea	Leaf (SAFE)	Profile XI (caryophyllane/*p*-menthane): α-pinene (12.3%) and *E*-caryophyllene 6.8%)	[64]
*P. guajava*	Lagos, Nigeria	Leaf (HD)	Profile XI (caryophyllane/*p*-menthane): limonene (42.1%) and *E*-caryophyllen (21.3%)	[65]
*P. guajava*	KwaZulu, Natal, South Africa	Leaf (HD)^a^ white fruit	Profile XII (caryophyllane/acyclic/cadinane): caryophyllene oxide (14.0%), *E*-caryophyllene (13.9%), 1H-cycloprop[e]azulene (11.7%), adamantane (9.5%), *E*-nerolidol (6.8%), and α-cubebene (6.7%)	[66]
*P. guajava*	Rio de Janeiro, RJ, Brazil	Leaf (HD)	Profile XIII (caryophyllane/eudesmane/cadinane): α-humulene (15.0%), *E*-caryophyllene (12.0%), β-selinene (11.0%), α-selinene (10.0%), cedr-8-(15)-en-9-α-ol (7.6%), and α-muurolol (5.6%)	[67]
*P. guajava*	Chenchou, Tunisia	Stems (HD)	Profile XIV (caryophyllane/germacrane/cadinane): germacrene D (16.8%), valerianol (10.6%), caryophyllene oxide (5.1%), and α-cadinol (5.0%)	[54]
*P. guajava*	Rio de Janeiro, RJ, Brazil	Leaf (HD)	Profile XIV (caryophyllane/eudesmane/cadinane): α-humulene (15.0%), *E*-caryophyllene (12.0%), α-selinene (10.0%), cedr-8(15)-en-9-α-ol (7.6%), and *epi*-α-muurolol (5.6%)	[104]
*P. guajava*	Rio de Janeiro, RJ, Brazil	Leaf (HD)	Profile XIV (caryophyllane/eudesmane/cadinane): caryophylla-4(12),8(13)-dien-5-β-ol (15.0%), α-humulene (13.0%), α-muurolol (9.6%), cedr-8-(15)-en-9-α-ol (7.4%), *E*-caryophyllene (7.2%), β-selinene (6.7%), humulene epoxide II (6.6%), and caryophyllene oxide (5.0%)	[67]
*P. guajava*	Rio de Janeiro, RJ, Brazil	Leaf (HD)	Profile XV (caryophyllane/eudesmane): α-humulene (37.0%), *E*-caryophyllene (24.0%), β-selinene (14.0%), *and* α-selinene (12.0%)	[67]
*P. guajava*	Nanning, China	Leaf (SD)	Profile XVI (*p*-menthane/aromadendrene): α-pinene (37.8%), 1,8-cineole (18.9%), globulol (6.8%), and cedrenol (5.6%)	[69]
*P. guajava*	Rio de Janeiro, RJ, Brazil	Leaf (HD)	Profile XVII (*p*-menthane/acyclic/caryophyllane/aromadendrene): α-pinene (25.5%), *E*-nerolidol (16.7%), *E*-caryophyllene (15.7%), δ-3-carene (8.8%), and cedran-8-ol (8.8%)	[70]
*P. guajava*	Alsharquia, Oman	Leaf (HD)	Profile XVII (*p*-menthane/acyclic/caryophyllane/aromadendrene): *E*-caryophyllene (33.5%), viridiflorene (13.0%), farnesene (11.7%), and limonene (9.8%)	[71]
*P. guajava*	Macas, Ecuador	Leaf (HD)	Profile XVIII (*p*-menthane): limonene (33.3%), α-pinene (29.5%), and carvotacetone acetate (8.2%)	[72]
*P. guajava*	El-Behera, Egypt	Leaf (HD)	Profile XVIII (*p*-menthane): limonene (54.7%) and 1, 8-cineole (32.1%)	[40]
*P. guajava*	Guira de Melena, Havana, Cuba	Fruit (SDE)	Profile XIX (*p*-menthane/others): limonene (8.3%), and 3-phenylpropyl acetate (6.2%)	[61]
*P. guajava*	Mauritius	Leaf (HD)	Profile XX (bisabolane/caryophyllane/*p*-menthane/cadinane): santalol (50.6%), caryophyllene oxide (15.4%), limonene (11.6%), and cycloisosativene (6.1%)	[73]
*P. guajava*	Monteverde, Costa Rica	Leaf (SDE)	Profile XXI (others/*p*-menthane/aromadendrene/acyclic): 2*E*-hexenal (28.4%), benzaldehyde (16.5%), 1,8-cineole (15.9%), globulol (10.3%), *E*-nerolidol (6.9%)	[74]
*P. guajava*	Saint-Denis, France	Fruit (HS-SPME)	Profile XXII (others): hexanal (65.9%), γ-butyrolactone (7.6%), 2*E*-hexenal (7.4%)	[49]
*P. guajava*	Guira de Melena, Havana, Cuba	Fruit (SDE)	Profile XXIII (others): 3*Z*-hexenyl acetate (5.0%)	[61]
*P. guajava*	Villahermosa, Mexico	Leaf (SD)	Profile XXIV (bisabolane/caryophyllane): β-bisabolene (19.2%), β-sesquiphellandrene (14.8%), *E*-caryophyllene (6.0%), *E*-γ-bisabolene (5.3%), and α-curcumene (5.1%)	[37]
*P. guajava*	Guira de Melena, Havana, Cuba	Fruit (SDE)	Profile XXV (others): *E*-cinnamyl acetate (5.6%)	[61]
*P. guajava*	Mimoso do Sul, ES, Brazil	Leaf (HD)	Profile XXVI: (caryophyllane/bisabolane/acyclic/*p*-menthane/cadinane/eudesmane/aromadendrene): *E*-caryophyllene (5.1–30.0%), α-humulene (2.0–24.4%), 14-hydroxy-*epi*-*E*-caryophyllene (1.3–19.3%), β-bisabolol (1.2–20.1%), *E*-nerolidol (0.5–19.9%), 14-hydroxy-*epi*-*E*-caryophyllene (0–14.7%), limonene (0.2–11.7%), γ-muurolene (1.5–6.4%), α-selinene (0.4–12.4%), β-selinene (0.5–13.3%), β-bisabolene (3.1–9.7%), hinesol (0.9–10.0%), *epi*-α-cadinol (0–6.4%), α-bisabolol (1.0–5.9%), selina-6-en-4-ol (0.6–9.1%), aromadendrene (0.3–7.4%), and 1,8-cineole (0.7–5.3%)	[24]
*P. guajava*	Linhares, ES, Brazil	Leaf (HD)	Profile XXVI (caryophyllane/bisabolane/acyclic/*p*-menthane/cadinane/eudesmane/aromadendrene): *E*-caryophyllene (5.1–32.3%), caryophyllene oxide (1.8–20.9%), α-humulene (1.7–19.9%), β-bisabolol (2.2–19.4%), *E*-nerolidol (2.1–13.7%), hinesol (3.2–12.4%), α-selinene (0.5–11.2%), β-selinene (0.5–12.8%), *epi*-α-cadinol (1.1–12.0%), limonene (0.1–11.0%), β-bisabolene (2.3–9.7%), α-bisabolol (0.5–7.3%), *epi*-β-cubenol (2.2.–7.1%), humulene epoxide (0.6–6.3%), selina-6-en-4-ol (3.3–6.1%), aromadendrene (3.1–5.6%), γ-muurolene (1.7–5.4%), and δ-cadinene (0.5–5.1%)	[24]
*P. guajava*	Abbottabad, Pakistan	Fruit (SD)	Profile XXVII (others): hexanol (13.9%), cinnamyl alcohol (10.9%), butanol (10.7%), 3-methyl glutaric anhydride (9.5%), hexene (7.7%), butanoic acid methyl ester (7.2%), and 3-hexenal (6.6%)	[75]
*P. guajava*	KwaZulu, Natal, South Africa	Leaf (HD)^a^ pink fruit	Profile XXVIII (others/caryophyllane): tetracyclo[6,3,2,0(2,5).0(1,8)] tridecan-9-ol,4,4-dimethyl (13.0%), *E*-caryophyllene (9.6%), 1H-cycloprop[e]azulene (8.1%)	[66]
*P. guineense*	Santarém, PA, Brazil	Leaf (HD)	Profile I (*p*-menthane): limonene (47.4%)	[21]
*P. guineense*	Curuçá, PA, Brazil	Leaf (HD)	Profile II (*p*-menthane/pinane): limonene (30.7%) and α-pinene (26.1%)	[21]
*P. guineense*	Curuçá, PA, Brazil	Leaf (HD)	Profile II (*p*-menthane/pinane): limonene (30.4%) and α-pinene (17.7%)	[21]
*P. guineense*	Curuçá, PA, Brazil	Leaf (HD)	Profile II (*p*-menthane/pinane): limonene (37.2%) and α-pinene (34.0%)	[21]
*P. guineense*	Curuçá, PA, Brazil	Leaf (HD)	Profile III (*p*-menthane/pinane): limonene (26.5%), α-pinene (13.7%), and α-copaene (7.2%)	[21]
*P. guineense*	Monte Alegre, PA, Brazil	Leaf (HD)	Profile IV (*p*-menthane/bisabolane): limonene (9.6%) and *epi*-β-bisabolol (6.5%)	[21]
*P. guineense*	Santarém, PA, Brazil	Leaf (HD)	Profile V (*p*-menthane/bisabolane): limonene (23.4%), *epi*-β-Bisabolol (9.5%), and β-bisabolene (6.4%),	[21]
*P. guineense*	Crato, CE, Brazil	Leaf (SD)	Profile VI (*p*-menthane/germacrane/pinane/elemane): 1,8-cineole (40.5%), β-eudesmol (19.5%), α-pinene (13.9%), β-pinene (8.6%), elemol (7.7%), and γ-eudesmol (5.2%)	[83]
*P. guineense*	Curuçá, PA, Brazil	Leaf (HD)	Profile VII (pinane/cadinane/caryophyllane): α-pinene (35.6%), α-copaene (8.1%), *E*-caryophyllene (6.1%), and muurola-4,10(14)-dien-1-β-ol (5.8%)	[21]
*P. guineense*	Santarém, PA, Brazil	Leaf (HD)	Profile VIII (pinane/*p*-menthane/caryophyllane): α-pinene (26.4%), limonene (14.0%), and *E*-caryophyllene (5.2%)	[21]
*P. guineense*	Monte Alegre, PA, Brazil	Leaf (HD)	Profile IX (bisabolane): β-bisabolene (8.9%) and α-curcumene (5.0%)	[21]
*P. guineense*	Ocozocoautla, Chiapas, Mexico	Leaf (SD)	Profile X (bisabolane/pinane/acyclic/*p*-menthane): β-bisabolene (13.2%), α-pinene (12.5%), *Z*-nerolidol (5.5%), β-sesquiphellandrene (5.2%), and limonene (5.1%)	[37]
*P. guineense*	Ponta de Pedras, PA, Brazil	Leaf (HD)	Profile XI (caryophyllane/*p*-menthane): *E*-caryophyllene (24.0%) and limonene (5.4%)	[21]
*P. guineense*	Boa Vista do Alto Alegre, RR, Brazil	Leaf/stems (HD)	Profile XII (bisabolane/*p*-menthane): β-bisabolol (17.4%), limonene (6.8%), and *epi*-α-bisabolol (6.7%)	[56]
*P. guineense*	Santarém, PA, Brazil	Leaf (HD)	Profile XIII (bisabolane): *epi*-β-bisabolol (18.1%) and β-bisabolol (5.6%)	[21]
*P. guineense*	Dourados, MS, Brazil	Leaf (HD)	Profile XIV (germacrane): spathulenol (80.7%)	[84]
*P. guineense*	La Palma, Cundinamarca, Colombia	Fruits (SDE)	Profile XV (caryophyllane/eudesmane/others): *E*-caryophyllene (8.6%), butanol (7.4%), ethyl butyrate (7.4%), and selin-11-en-4α-ol (5.9%)	[85]
*P. guineense*	La Palma, Cundinamarca, Colombia	Fruits (HS-SPME)	Profile XVI (others): ethyl butyrate (30.3%) and ethyl hexanoate (23.8%)	[85]
*P. laruotteanum*	Brasilia, Brazil	Leaves (HD)	Profile I (*p*-menthane/pinane): *p*-cymene (24.8%), 1,8-cineole (19.2%), α-pinene (13.4%), and terpinen-4-ol (6.3%)	[86]
*P. laruotteanum*	Brasilia, Brazil	Leaves (HD)	Profile II (*p*-menthane/pinane): *p*-cymene (19.4%), γ-terpinene (14.0%), α-pinene (11.6%), limonene (10.2%), 1,8-cineole (6.9%), terpinen-4-ol (5.8%), terpinolene (5.1%)	[86]
*P. laruotteanum*	Brasilia, Brazil	Leaves (HD)	Profile III (*p*-menthane/pinane): *p*-cymene (34.8%), 1,8-cineole (12.5%), α-pinene (9.2%), limonene (7.9%), γ-terpinene (6.9%), and α-terpineol (6.0%)	[86]
*P. montanum*	West region, Cuba	Leaf (HD)	Profile *p*-menthane/pinane: 1,8-cineole (46.9%), α-terpineol (9.2%), and α-pinene (8.9%)	[101]
*P. myrsinites*	Anápolis, GO, Brazil	Leaf (HD)	Profile I (caryophyllane): *E*-caryophyllene (31.0%), α-humulene (12.3%), and caryophyllene oxide (7.3%)	[88]
*P. myrsinites*	Chapada das Mesas National Park, MA, Brazil	Leaf (HD)	Profile II (caryophyllane): *E*-caryophyllene (26.1%), 𝛼-humulene (23.9%), caryophyllene oxide (10.1%), humulene epoxide II (6.4%), and Caryophylla-4(12),8(13)-dien- 5-β-ol (5.7%)	[89]
*P. myrsinites*	Brasilia, Brazil	Leaf/flower (HD)	Profile III (caryophyllane/acyclic): caryophyllene oxide (26.1%), humulene epoxide II (8.8%), *E*-caryophyllene (7.4%), Z-caryophyllene (5.4%), and myrcene (5.4%)	[90]
*P. myrtoides*	Brasilia, Brazil	Leaf (HD)	Profile I (caryophyllane/acyclic) *E*-caryophyllene (22.4%), caryophyllene oxide (19.7%), α-humulene (8.4%), and myrcene (5.4%)	[23]
*P. myrtoides*	Chapada, do Araripe, CE, Brazil	Leaf (HD) ^b^	Profile II (*p*-menthane/germacrane/pinane/elemane) 1,8-cineole (29.5–48.1%), α-eudesmol (11.7–20.0%), α-pinene (5.0–12.8%), elemol (3.3–6.7%), and γ-eudesmol (2.5–5.8%)	[93]
*P. myrtoides*	Rio verde, GO, Brazil	Leaf (HD)	Profile III (caryophyllane/cadinane/bisabolane): *E*-caryophyllene (30.9%), α-humulene (15.9%), α-copaene (7.8%), caryophyllene oxide (7.3%), and α-bisabolol (7.3%)	[91]
*P. myrtoides*	Alegre, ES, Brazil	Leaf (HD)	Profile III (caryophyllane/cadinane/bisabolane): *E*-caryophyllene (19.4%), α-bisabolol (10.4%), α-humulene (10.4%), α-copaene (6.3%), and caryophyllene oxide (5.3%)	[22]
*P. parvifolium*	Pinar del Río, Cuba	Leaf (HD)	viridiflorol (31.9%), α-terpineol (8.2%), cubenol (7.3%), borneol (7.2%), *epi*-α-muurolol (6.6%), and *trans*-sabinol (5.5%)	[42]
*P. rotundatum*	Pinar del Rio, Cuba	Leaf/Stalks (SDE)	1,8-cineole (28.0%), α-pinene (18.3%), α-terpineol (9.2%), *E*-nerolidol (8.7%), and linalool (5.1%)	[102]
*P. salutare*	Punta Espinillo, Montevideo, Uruguay	Leaf/Twig (SD)	Profile I (*p*-menthane/acyclic): 1,8-cineole (31.1%), linalool (11.5%), and α-terpineol (7.0%)	[96]
*P. salutare*	Punta Espinillo, Montevideo, Uruguay	Leaf/Twig (SD)	Profile I (*p*-menthane/acyclic): 1,8-cineole (36.6%), linalool (12.4%), and α-terpineol (6.7%)	[96]
*P. salutare*	Crato, CE, Brazil	Leaf (SD)	Profile II (*p*-menthane/germacrane): 1,8-cineole (63.3%), *p*-cymene (14.1%), α-terpinyl acetate (7.2%), and β-eudesmol (8.8%)	[83,97]
*P. salutare*	Chapada do Araripe, CE, Brazil	Leaf (HD) ^b^	Profile III (*p*-menthane/cadinane/acyclic): *p*-cymene (5.1–17.8%), terpinolene (6.9–17.0%), γ-terpinene (10.3–17.1%), *epi*-α-cadinol (10.4–12.8%), linalool (4.7–7.3%), and δ-cadinene (3.8–5.3%)	[25]
*P. salutare*	Pinar del Rio, Cuba	Leaf (HD)	Profile IV (caryophyllane/bisabolane/eudesmane/pinane): caryophyllene oxide (39.8%), *ar*-turmerone (17.3%), β-gurjunene (6.7%), β-selinene (6.0%), and α-pinene (5.6%)	[98]
*P. sartorianum*	Pinar del Rio, Cuba	Leaf (HD)	Profile I (*p*-menthane/pinane): limonene (43.0%), α-pinene (39.5%), and β-pinene (5.6%)	[99]
*P. sartorianum*	Guadalajara, Mexico	Leaf (SD)	Profile II (pinane/caryophyllane/*p*-menthane/acyclic): α-pinene (16.7%), *E*-caryophyllene (12.4%), α-phellandrene (9.8%), and *Z*-nerolidol (5.2%)	[37]
*P. striatulum*	Boa Vista, RR, Brazil	Fruits (HD)	Profile I (pinane/caryophyllane/cadinane/aromadendrene): α-pinene (12%), α-humulene (10.4%), α-copaene (7.1%), globulol (5.7%), and aromadendrene (5.1%)	[100]
*P. striatulum*	Carolina, MA, Brazil	Leaf/stems (HD)	Profile II (caryophyllane/eudesmane) *E*-caryophyllene (28.6%), α-selinene (7.7%), caryophyllene oxide (7.6%), β-selinene (7.4%), selin-11-en-4-α-ol (6.0%)	[56]
*P. rufum*	Rio de Janeiro, RJ, Brazil	Leaf (HD)	*E*-caryophyllene (21.0%), α-pinene (14.0%), γ-eudesmol (8.5%), 1,8-cineole (8.4%), α-eudesmol (8.2%), and β-eudesmol (6.8%)	[104]

^a^ = doubtful analysis, unknown RI values, ^b^ = seasonal study, SD = steam distillation, HD = hydrodistillation, SPME = solid phase micro extraction, HS-SPME = headspace—solid phase micro extraction, SDE = simultaneous steam distillation-solvent extraction, SAFE = solvent-assisted flavor evaporation, SE **=** solvent extraction.

## Appendix B

**Table A2:** Species of *Psidium*: essential oil biological activity

Species	Occurrence	Plant Part/ Extraction Type	Primary Components (>5%)	Essential Oil Bioactivity	Ref.
*P. brownianum*	Crato, CE, Brazil	Leaf (HD)	β-eudesmol (27.1%), 1,8-cineole (24.7%), α-elemol (11.8%), α-pinene (11.4%), guaiol (9.1%), and β-pinene (8.4%)	Antinociceptive effect (doses 100 and 200 mg/kg)	[103]
*P. cattleyanum*	Pelotas, RS, Brazil	Leaf (SD)	*E*-caryophyllene (59.6%), caryophyllene oxide (18.2%), and Z-caryophyllene (6.4%)	Antioxidant *in vitro*, DPPH assay (inactive at 10–500 mg/mL); Antioxidant *in vitro,* ABTS assay (inactive at 10–500 mg/mL); Antioxidant *in vitro*, linoleic acid oxidation assay (IC_50_ 56.41 μg/mL) Toxicity, mouse model oral administration (LD_50_ > 500mg/Kg) Antifungal, broth microdilution method (*Candida albicans*, MIC 166.7 µg/mL; *Candida lipolytica,* MIC 125 µg/mL; *Candida guilhermondii,* MIC 125 µg/mL; *Candida parapsilosis,* MIC 104.2 µg/mL; *Trichosporon asahii,* MIC 41.76 µg/mL)	[38]
*P. cattleyanum*	Alegre, ES, Brazil	Leaf (HD)	*E*-caryophyllene (23.4%), caryophyllene oxide (11.5%), and α-pinene (11.3%)	Phytotoxic, dose 3000 µg/mL (*Lactuca sativa*, Germination inhibition 74.6%, Germination Speed Index 3.4 mm; *Sorghum bicolor*, Germination inhibition 92.6%, Germination Speed Index, 6.9 mm)	[22]
*P. cattleyanum*	El-Behera, Egypt	Leaf (HD)	*E*-caryophyllene (28.8%), α-pinene (28.0%), myrcene (13.4%), and *trans*-β-ocimene (5.3%)	Antibacterial, disk diffusion method (*Neisseria gonorrhoeae*, MIC 13.01 µg/mL)	[40]
*P. cattleyanum*	Pelotas, RS, Brazil	Fruit (HD)	*E*-caryophyllene (22.5%), *neo*-intermedeol (14.2%), β-selinene (10.1%), *trans*-β-guaiene (9.1%), and α-humulene (7.5%)	Antioxidant, DPPH assay on TLC plate (1:250 dilution)	[41]
*P. cattleyanum*	Limeira, SP, Brazil	Leaf (HD)	Viridiflorol (17.9%), *E*-caryophyllene (11.8%), 1,8-cineole (10.8%), β-selinene (8.6%), α-humulene (6.0%), and aromadendrene (5.0%)	Antibacterial, broth microdilution assay (*Porphyromonas gingivalis*, MIC 20 µg/mL; *Prevotella nigrescens*, MIC 62.5 µg/mL; *Fusobacterium nucleatum*, MIC 12.5 µg/mL; *Bacteroides fragilis*, MIC 12.5 µg/mL; *Actinomyces naeslundii*, MIC 50 µg/mL; *Peptostreptococcus anaerobius*, MIC 62.5 µg/mL; *Aggregatibacter actinomycetemcomitans*, MIC 6.25 µg/mL)	[44]
*P. friedrichsthalianum*	Alegre, ES, Brazil	Leaf (HD)	*E*-caryophyllene (24.6%), caryophyllene oxide (10.6%), α-humulene (9.2%), α-copaene (5.9%)	Phytotoxic, dose 375 µg/mL (*Lactuca sativa*, Germination inhibition 92.8%, Germination Speed Index 5.7 mm; *Sorghum bicolor*, Germination inhibition 91.7%, Germination Speed Index, 8.4 mm)	[22]
*P. gaudichaudianum*	Alegre, ES, Brazil	Leaf (HD)	*E*-caryophyllene (17.0%), limonene (16.2%), α-pinene (8.4%), caryophyllene oxide (7.5%), and α-humulene (5.8%)	Phytotoxic, dose 1500 µg/mL (*Lactuca sativa*, Germination inhibition 90.7%, Germination Speed Index 5.2 mm; *Sorghum bicolor*, Germination inhibition 91.1%, Germination Speed Index, 8.1 mm)	[22]
*P. guajava*	Mauritius	Leaf (HD)	Caryophyllene oxide (15.4%) and limonene (11.6%)	Antioxidant, DPPH assay (IC_50_ 5.19 µg/mL); Antioxidant, ABTS assay (IC_50_ 3.09 µg/mL); Antioxidant, XO assay (IC_50_ 2.51 µg/mL); Antioxidant, OH assay (IC_50_ 1.90 µg/mL); Antioxidant, NO assay (IC_50_ 2.71 µg/mL); Antioxidant, ORAC assay (0.275 TE/gEO); Antioxidant, FRAP assay (44.41 µmol Fe^+2^/mg OE);	[62]
*P. guajava*	Mauritius	Leaf (HD)	Santalol (50.6%), caryophyllene oxide (15.4%), limonene (11.6%), and cycloisosativene (6.1%)	Antibacterial, disc diffusion assay (*Enterococcus faecalis,* 16.5 mm; *Escherichia coli,* 19.4 mm, Methicillin Resistant *Staphylococcus aureus*, 7.6 mm; *Pseudomonas aeruginosa,* 8.0 mm; *Staphylococcus aureus,* 18.6 mm; *Staphylococcus epidermidis,* 18.2 mm)	[73]
*P. guajava*	Macas, Ecuador	Leaf (HD)	Limonene (33.3%), α-pinene (29.5%), and carvotacetone acetate (8.2%)	Antifungal, disk diffusion assay (*Candida albicans,* MIC 0.14 mg/mL; *Rhodotorula glutinis,* MIC 0.09 mg/mL; *Schizosaccharomyces pombe,* MIC 0.09 mg/mL; *Saccharomyces cerevisiae,* MIC 0.06 mg/mL; *Yarrowia lypolitica,* MIC 0.23 mg/mL)	[72]
*P. guajava*	Bozhou, Anhui, China	Leaf (HD)	*E*-caryophyllene (26.4%), β-bisabolene (5.23%), and γ-gurjunene (15.2%)	Antioxidant activity: DPPH assay (IC_50_ 23.39 mg/mL); ABTS assay (IC_50_ 18.34 mg/mL); FRAP assay (6.57 mmol Vc/g DM) Antimicrobial, disc diffusion assay (*Escherichia coli,* 9.24 mm; *Alcaligenes faecalis,* 11.46 mm; *Bacillus aryabhattai,* 18.18 mm; *Arthrobacter creatinolyticus,* 11.35 mm; *Bacillus megaterium,* 19.18 mm; *Bacillus subtilis,* 17.69 mm; *Saccharomyces cerevisiae,* 18.76 mm; *Rhodotorula* sp., 19.35 mm)	[53]
*P. guajava*	Panyu, Guangdong, China	Leaf (HD)	*E*-caryophyllene (25.7%), calamenene (7.4%), γ-gurjunene (9.5%%), and *epi*-α-cadinol (6.4%)	Antioxidant activity: DPPH assay (IC_50_ 18.52 mg/mL); ABTS assay (IC_50_ 13.12 mg/mL); FRAP assay (9.13 mmol Vc/g DM) Antimicrobial, disc diffusion assay (*Escherichia coli,* 10.54 mm; *Alcaligenes faecalis,* 16.54 mm; *Bacillus aryabhattai,* 23.15 mm; *Arthrobacter creatinolyticus,* 15.27 mm; *Bacillus megaterium,* 22.98 mm; *Bacillus subtilis,* 19.34 mm; *Saccharomyces cerevisiae,* 20.13 mm; *Rhodotorula* sp., 26.36 mm)	[53]
*P. guajava*	Jiangmen, Guangdong, China	Leaf (HD)	*E*-caryophyllene (25.0%), calamenene (7.1%), γ-gurjunene (9.5%), and *epi*-α-cadinol (6.0%)	Antioxidant activity: DPPH assay (IC_50_ 19.42 mg/mL); ABTS assay (IC_50_ 15.31 mg/mL); FRAP assay (7.68 mmol Vc/g DM) Antimicrobial, disc diffusion assay (*Escherichia coli,* 11.23 mm; *Alcaligenes faecalis,* 14.14 mm; *Bacillus aryabhattai,* 22.79 mm; *Arthrobacter creatinolyticus,* 14.97 mm; *Bacillus megaterium,* 21.45 mm; *Bacillus subtilis,* 18.89 mm; *Saccharomyces cerevisiae,* 21.23 mm; *Rhodotorula* sp., 26.71 mm)	[53]
*P. guajava*	Shijiazhuang, Hebei, China	Leaf (HD)	*E*-caryophyllene (27.4%) and γ-gurjunene (13.5%)	Antioxidant activity: DPPH assay (IC_50_ 23.44 mg/mL); ABTS assay (IC_50_ 19.13 mg/mL); FRAP assay (6.92 mmol Vc/g DM) Antimicrobial, disc diffusion assay (*Escherichia coli,* 8.76 mm; *Alcaligenes faecalis,* 9.26 mm; *Bacillus aryabhattai,* 17.34 mm; *Arthrobacter creatinolyticus,* 10.24 mm; *Bacillus megaterium,* 17.39 mm; *Bacillus subtilis,* 18.27 mm; *Saccharomyces cerevisiae,* 18.69 mm; *Rhodotorula* sp., 19.35 mm)	[53]
*P. guajava*	Anguo, Hebei, China	Leaf (HD)	*E*-caryophyllene (24.4%) and γ-gurjunene (12.7%)	Antioxidant activity: DPPH method (IC_50_ 24.07 mg/mL); ABTS method (IC_50_ 20.34 mg/mL); FRAP method (6.91 mmol Vc/g DM) Antimicrobial, disc diffusion method (*Escherichia coli,* 8.35 mm; *Alcaligenes faecalis,* 9.78 mm; *Bacillus aryabhattai,* 17.26 mm; *Arthrobacter creatinolyticus,* 10.57 mm; *Bacillus megaterium,* 17.08 mm; *Bacillus subtilis,* 18.34 mm; *Saccharomyces cerevisiae,* 18.76 mm; *Rhodotorula* sp., 19.78 mm)	[53]
*P. guajava*	Taipei, Taiwan	Leaf (HD)	*E*-caryophyllene (17.2), γ-gurjunene (9.3%), *epi*-α-cadinol (10.0%), and calamenene (6.7%)	Antioxidant activity: DPPH assay (IC_50_ 31.12 mg/mL); ABTS assay (IC_50_ 24.15 mg/mL); FRAP assay (2.36 mmol Vc/g DM) Antimicrobial, disc diffusion assay (*Escherichia coli,* 7.89 mm; *Alcaligenes faecalis,* 10.23 mm; *Bacillus aryabhattai,* 15.34 mm; *Arthrobacter creatinolyticus,* 9.12 mm; *Bacillus megaterium,* 16.89 mm; *Bacillus subtilis,* 17.02 mm; *Saccharomyces cerevisiae,* 16.89 mm; *Rhodotorula* sp., 20.23 mm)	[53]
*P. guajava*	Hangzhou, Zhejiang, China	Leaf (HD)	*E*-caryophyllene (31.4%) and γ-gurjunene (14.0%),	Antioxidant activity: DPPH assay (IC_50_ 20.36 mg/mL); ABTS assay (IC_50_ 19.39 mg/mL); FRAP assay (7.12 mmol Vc/g DM) Antimicrobial, disc diffusion assay (*Escherichia coli,* 8.98 mm; *Alcaligenes faecalis,* 11.13 mm; *Bacillus aryabhattai,* 18.79 mm; *Arthrobacter creatinolyticus,* 10.79 mm; *Bacillus megaterium,* 19.09 mm; *Bacillus subtilis,* 18.01 mm; *Saccharomyces cerevisiae,* 17.11 mm; *Rhodotorula* sp., 18.34 mm	[53]
*P. guajava*	Tainan, Taiwan	Leaf (HD)	*E*-caryophyllene (21.4%), γ-gurjunene (9.2%), *epi*-α-cadinol (7.8%), and calamenene (6.6%)	Antioxidant activity, DPPH assay (IC_50_ 33.71 mg/mL); Antioxidant activity, ABTS assay (IC_50_ 25.35 mg/mL), Antioxidant activity, FRAP assay (2.29 mmol Vc/g DM); Antimicrobial, disc diffusion assay (*Escherichia coli,* 7.90 mm; *Alcaligenes faecalis,* 10.15 mm; *Bacillus aryabhattai,* 15.76 mm; *Arthrobacter creatinolyticus,* 9.34 mm; *Bacillus megaterium,* 17.02 mm; *Bacillus subtilis,* 18.10 mm; *Saccharomyces cerevisiae,* 18.98 mm; *Rhodotorula* sp., 19.90 mm)	[53]
*P. guajava*	Meizhou, Guangdong, China	Leaf (HD)	*E*-caryophyllene (25.0%), γ-gurjunene (9.5%), *epi*-α-cadinol (6.1%), and calamenene (7.8%)	Antioxidant activity: DPPH assay (IC_50_ 20.26 mg/mL); ABTS assay (IC_50_ 16.18 mg/mL); FRAP assay (7.34 mmol Vc/g DM) Antimicrobial, disc diffusion assay (*Escherichia coli,* 10.38 mm; *Alcaligenes faecalis,* 14.65 mm; *Bacillus aryabhattai,* 21.97 mm; *Arthrobacter creatinolyticus,* 14.65 mm; *Bacillus megaterium,* 21.22 mm; *Bacillus subtilis,* 18.97 mm; *Saccharomyces cerevisiae,* 20.57 mm; *Rhodotorula* sp., 25.98 mm)	[53]
*P. guajava*	Kathmandu, Nepal	Leaf (HD)	*E*-nerolidol (35.6%), *E*-caryophyllene (15.8%), (2*Z*,6*E*)-farnesol (6.7%), and ledol (5.5%)	Larvicidal activity against *Chaoborus plumicornis* (LC_50_ 63.3 μg/mL); Insecticidal activity against *Drosophila melanogaster* (LC_50_ 327 μg/mL) Nematicidal activity against *Caenorhabditis elegans* with (LC50 of 142 μg/mL).	[51]
*P. guajava*	Alsharquia, Oman	Leaf (HD)	*E*-caryophyllene (33.5%), viridiflorene (13.0%), farnesene (11.7%), and limonene (9.8%)	Antibacterial activity, disc diffusion assay (*Enterococcus faecales,* 6 mm; *Staphylococcus aureus,* 9 mm; *Haemophilus influenzae,* 12 mm; *Pseudomonas aeruginosa,* 6 mm; *Escherichia coli,* 13 mm)	[71]
*P. guajava*	El-Behera, Egypt	Leaf (HD)	limonene (54.7%) and 1, 8-cineole (32.1%)	Antibacterial activity, disc diffusion assay (*Staphylococcus aureus*, MIC 6.75 µg/mL)	[40]
*P. guajava*	Lucknow, Uttar Pradesh, India	senescent leaves (HD)	Limonene (29.1%), *E*-caryophyllene (15.7%), caryophyllene oxide (8.8%), caryophylla-4(12),8(13)-dien-5β-ol (6.5%)	Antibacterial activity, disc diffusion and microdilution broth assays (*Staphylococcus aureus* methicillin-resistant, 65 µg/mL; *S. aureus,* 65–261 µg/mL; *Staphylococcus epidermidis*, 130 µg/mL; *S. epidermidis* methicillin-resistant, 65 µg/mL; *Mycobacterium smegmatis*, 261 µg/mL) Antifungal activity, disc diffusion and microdilution broth assays (*Candida krusei*, 16.71 mg/mL)	[63]
*P. guajava*	Parnaíba, PI, Brazil	Leaf (SD)	*E*-caryophyllene (39.0%), β-selinene (9.7%), α-selinene (9.7%), 1,8-cineole (6.9%), and aromadendrene (6.3%)	Acaricidal activity (females of *RhipicephalusMicroplus,* adult immersion test, 99.95% of efficiency on engorged at 12.5 mg/L; larval packet test, larvae mortality 5.8% at 12.5 mg/L)	[58]
*P. guajava*	Rio de Janeiro, RJ, Brazil	Leaf (HD)	α-humulene (15.0%), *E*-caryophyllene (12.0%), β-selinene (11.0%), α-selinene (10.0%), cedr-8-(15)-en-9-α-ol (7.6%), and α-muurolol (5.6%)	Anti-inflammatory activity, Pleurisy induced by lipopolysaccharide model (inhibition in migration of eosinophils 76% at 100 mg/kg)	[67]
*P. guajava*	Rio de Janeiro, RJ, Brazil	Leaf (HD)	α-humulene (37.0%), *E*-caryophyllene (24.0%), β-selinene (14.0%), and α-selinene (12.0%)	Anti-inflammatory activity, Pleurisy induced by lipopolysaccharide model (inhibition in migration of eosinophils 67% at 100 mg/kg)	[67]
*P. guajava*	Rio de Janeiro, RJ, Brazil	Leaf (HD)	caryophylla-4(12),8(13)-dien-5-β-ol (15.0%), α-humulene (13.0%), α-muurolol (9.6%), cedr-8-(15)-en-9-α-ol (7.4%), *E*-caryophyllene (7.2), β-selinene (6.7%), humulene epoxide II (6.6%), and caryophyllene oxide (5.0%)	Anti-inflammatory, Pleurisy induced by LPS model (migration inhibition of eosinophils 74% at 100 mg/kg)	[67]
*P. guajava*	Alegre, ES, Brazil	Leaf (HD)	*E*-caryophyllene (26.6%), selin-11-en-4α-ol (6.7%), caryophyllene oxide (15.5%), aromadendrene epoxide (8.1%), β-selinene (7.6%), and α-selinene (6.5%)	Larvicidal activity against *Aedes aegypti* (LC_50_ 39.48 μg/mL; LC_90_ 57.34 μg/mL)	[55]
*P. guajava*	Alegre, ES, Brazil	Leaf (HD)	*E*-caryophyllene (7.6%), β-bisabolol (19.5%), limonene (17.8%), 1,8-cineole (5.1%), and *E*-nerolidol (6.9%)	Larvicidal activity against *Aedes aegypti* (LC_50_ 51.11 μg/mL; LC_90_ 71.56 μg/mL)	[55]
*P. guajava*	Alegre, ES, Brazil	Leaf (HD)	*E*-caryophyllene (9.4%), β-bisabolol (15.1%), limonene (6.5%), α-humulene (16.5%), *E*-nerolidol (7.4%), β-bisabolene (6.3%), and humulene epoxide II (6.0%)	Larvicidal activity against *Aedes aegypti* (LC_50_ 53.47 μg/mL; LC_90_ 73.84 μg/mL)	[55]
*P. guajava*	Alegre, ES, Brazil	Leaf (HD)	*E*-caryophyllene (19.4%), selin-11-en-4α-ol (7.4%), caryophyllene oxide (16.6%), aromadendrene epoxide (9.2%), 1,8-cineole (8.4%), and β-selinene (5.6%)	Larvicidal activity against *Aedes aegypti* (LC_50_ 63.35 μg/mL; LC_90_ 82.44 μg/mL)	[55]
*P. guajava*	Alegre, ES, Brazil	Leaf (HD)	*E*-caryophyllene (10.2%), selin-11-en-4α-ol (16.7%), caryophyllene oxide (8.0%), aromadendrene epoxide (9.5%), 1,8-cineole (7.6%), *epi*-α-cadinol (7.9%), β-selinene (8.2%), *epi*-cubenol (6.7%), and α-selinene (6.3%)	Larvicidal activity against *Aedes aegypti* (LC_50_ 64.25 μg/mL; LC_90_ 86.00 μg/mL)	[55]
*P. guajava*	Rio de Janeiro, RJ, Brazil	Leaf (HD)	α-pinene (25.5%), *E*-nerolidol (16.7%), *E*-caryophyllene (15.7%), δ-3-carene (8.8%), and cedran-8-ol (8.8%)	Inseticidal activity (*Tribolium castaneum*, Fumigation LC_50_ 6.1 µg/L air after 24 h of treatment and < 2 µg/L air after 72 h Inseticidal activity (*Culex pipiens,* Fumigation LC_50_ > 50 µg/L) Larvicidal activity (*Culex pipiens,* Fumigation LC_50_ > 100 µg/L)	[70]
*P. guajava*	Abbottabad, Pakistan	Fruit (SD)	Hexanol (13.9%), cinnamyl alcohol (10.9%), Butanol (10.7%), 3-methyl glutaric anhydride (9.5%), hexene (7.7%), butanoic acid methyl ester (7.2%), and 3-hexenal (6.6%)	Vasorelaxant effect, rabbit aorta preparations against pre-concentrations of K^+^ (EC_50_ 5.52 mg/mL); Vasorelaxant effect, rabbit aorta preparations against phenylephrine (EC_50_ 6.23 mg/mL); Spasmolytic effect, isolated rabbit jejunum against contractions spontaneous (EC_50_ 0.84 mg/mL) Spasmolytic effect, isolated rabbit jejunum against induced contractions by K^+^ (EC_50_ 0.71 mg/mL)	[75]
*P. guajava*	Cairo, Egypt	Fruit (HD)	*E*-caryophyllene (17.6%), limonene (11.0%), α-selinene (6.6%), and β-selinene (6.4%)	Antioxidant activity: DPPH assay (IC_50_ 8.11 mg/mL); deoxyribose degradation (IC_50_ 42.78 μg/mL); Anti-inflammatory activity: inhibition of 5-lipoxygenase (IC_50_ 49.76 μg/mL); Cytotoxic activity (HepG2 hepatic cancer, IC_50_ 196.45 µg/mL; MCF-7 breast cancer, IC_50_ 544.38 µg/mL)	[57]
*P. guajava*	Cairo, Egypt	Leaf (HD)	*E*-caryophyllene (16.9%), selin-7(11)-en-4-α-ol (8.3%), α-selinene (6.5%), β-selinene (6.3%), 1,8-cineole (5.4%), and δ-cadinene (5.3%)	Antioxidant activity: DPPH assay (IC_50_ 3.59 mg/mL); deoxyribose degradation (IC_50_ 12.64 μg/mL); Anti-inflammatory activity: inhibition of 5-lipoxygenase (IC_50_ 32.53 μg/mL); Cytotoxic activity: HepG2 hepatic cancer, IC_50_ 130.69 µg/mL; MCF-7 breast cancer, IC_50_ 351.00 µg/mL)	[57]
*P. guineense*	Dourados, MS, Brazil	Leaf (HD)	Spathulenol (80.7%)	Antioxidant activity: DDPH assay (IC_50_ 63.08 µg/mL); ABTS assay (IC_50_ 780.13 µg/mL); MDA assay (IC_50_ 37.91 µg/mL) Anti-inflammatory activity: carrageenan-induced mice paw oedema model, inhibition of 59.46% after second and fourth hour at 300 mg/kg); pleurisy model, reduction in the increase in total leukocytes of 45.33% at 30 mg/kg and 77.70% at 100 mg/kg); reduction in the rise in protein levels of 49.72%, at 30 mg/kg, and 78.40%, at 100 mg/kg;Cytotoxic activity: U251 glioma, GI_50_ 9.84 µg/mL; MCF-7 breast, GI_50_ 7.90 µg/mL; NCI/ADR-RES ovarian expressing the phenotype of multiple drug resistance, GIC_50_ 9.25 µg/mL; 786–0 renal, GI_50_ 2.57 µg/mL; NCI-H460 lung, GI_50_ 4.57 µg/mL; PCO-3 prostate, GI_50_ 9.18 µg/mL; OVCAR-3 ovarian, GI_50_ 0.89 µg/mL; HT-29 colon, GI_50_ 5.62 µg/mL; K-562 leukemia, GI_50_ 5.03 µg/mL; HaCaT keratinocytes, GI_50_ 7.98 µg/mL)	[84]
*P. myrsinites*	Anápolis, GO, Brazil	Leaf (HD)	*E*-caryophyllene (31.0%), α-humulene (12.3%), and caryophyllene oxide (7.3%)	Larvicidal activity against *Artemia salina* (LC_50_ 95.3 µg/mL)	[88]
*P. myrsinites*	Chapada das Mesas National Park, MA, Brazil	Leaf (HD)	*E*-caryophyllene (26.1%), 𝛼-humulene (23.9%), caryophyllene oxide (10.1%), humulene epoxide II (6.4%), and Caryophylla-4(12),8(13)--ien-5-β-ol (5.7%)	Larvicidal activity against *Aedes aegypti* (LC_50_ 292 mg/mL)	[89]
*P. myrtoides*	Chapada, do Araripe, CE, Brazil	Leaf (HD) ^b^	1,8-cineole (29.5–48.1%), α-eudesmol (11,7–20.0%), α-pinene (5.0–12.8%), elemol(3.3–6.7%), and γ-eudesmol (2.5–5.8%)	Antifungal activity: broth microdilution assay (*Candida albicans,* MFC 1.02–4.10 µg/mL, IC_50_ 103.3–963.8 µg/mL; *C. krusei,* MFC 8.19–16.38 µg/mL, IC_50_ 1235.9–3564.5 µg/mL; *C. tropicalis,* MFC > 16.384 µg/mL, IC_50_ 1671.1–2535.1 µg/mL)	[93]
*P. myrtoides*	Rio verde, GO, Brazil	Leaf (HD)	*E*-caryophyllene (30.9%), α-humulene (15.9%), α-copaene (7.8%), caryophyllene oxide (7.3%), and α-bisabolol (7.3%)	Antibacterial activity, broth microdilution assay: *Streptococcus mitis,* MIC100 μg/mL; *S. sanguinis,* MIC 100 μg/mL; *S. sobrinus,* MIC 250 μg/mL; *S. salivarius,* MIC 250 μg/mL; *S. mutans* MIC, 62.5 μg/mL; Cytotoxic activity: MCF-7 human breast adenocarcinoma, IC_50_ 254.5 μg/mL; HeLa, human cervical adenocarcinoma, IC_50_ 324.2 μg/mL; M059J human glioblastoma, IC_50_ 289.3 μg/mL)	[91]
*P. myrtoides*	Alegre, ES, Brazil	Leaf (HD)	*E*-caryophyllene (19.4%), α-bisabolol (10.4%), α-humulene (10.4%), α-copaene (6.3%), and caryophyllene oxide (5.3%)	Phytotoxic activity (dose 3000 µg/mL): *Lactuca sativa*, Germination inhibition 47.4%, Germination Speed Index 3.4 mm; *Sorghum bicolor*, Germination inhibition 90.4%, Germination Speed Index, 7.8 mm)	[22]
*P. salutare*	Crato, CE, Brazil	Leaf (SD)	1,8-cineole (63.3%), *p*-cymene (14.1%), α-terpinyl acetate (7.2%), and β-eudesmol (8.8%)	Antinociceptive effect (dose 400 mg/kg in mouse model)	[83,97]
*P. salutare*	Chapada do Araripe, CE, Brazil	Leaf (HD) ^b^	*p*-cymene (5.1–17.8%), terpinolene (6.9–17.0%), γ-terpinene (10.3–17.1%), *epi*-α-cadinol (10.4–12.8%), linalool (4.7–7.3%), and δ-cadinene(3.8–5.3%)	Antifungal activity: *Candida albicans,* MFC 1.02–4.10 µg/mL; *Candida krusei,* MFC 8.19–16.38 µg/mL; *Candida tropicalis,* MFC > 16.38 mg/mL)	[25]
*P. striatulum*	Boa Vista, RR, Brazil	Fruits (HD)	α-pinene (12%), α-humulene (10.4%), α-copaene (7.1%), globulol (5.7%), and aromadendrene (5.1%)	Antibacterial activity: *Staphylococcus aureus,* IC_50_ 28.62 μg/mL; *Bacillus cereus,* IC_50_ 24.74 μg/mL; *Salmonella typhimurium,* IC_50_ 18.69 μg/mL); Enzyme acetylcholinesterase, inhibition 44.42%)	[100]
*P. rufum*	Rio de Janeiro, RJ, Brazil	Leaf (HD)	*E*-caryophyllene (21.0%), α-pinene (14.0%), γ-eudesmol (8.5%), 1,8-cineole (8.4%), α-eudesmol (8.2%), and β-eudesmol (6.8%)	Anti-inflammatory activity: zymosan induced inflammatory model (reduction in eosinophil migration 70% at 100 mg/kg), in vitro nitric oxide production (moderate effect, 51% at 100 mg/kg)	[104]

^a^ = doubtful analysis, unknown RI values, ^b^ = seasonal study, SD = steam distillation, HD = hydrodistillation, SPME = solid phase micro extraction, HS-SPME = headspace—solid phase micro extraction, SDE = simultaneous steam distillation-solvent extraction, SAFE = solvent-assisted flavor evaporation, SE = solvent extraction IC_50_ = median inhibitory concentration, MIC = minimum inhibitory concentration, MMC = minimum microbicide concentration, LC_50_ = median lethal concentration; MFC = minimum fungicide concentration, MDA = malondialdehyde, TBARS = thiobarbituric acid reactive species, NO = nitric oxide radical scavenging assay; XO = xanthine oxidase assay; ORAC = oxygen radical absorbing capacity assay, FRAP = ferric reducing antioxidant power, DPPH = 2,2-diphenyl-1-picrylhydrazyl.
